# Regulation and impact of cardiac lymphangiogenesis in pressure-overload-induced heart failure

**DOI:** 10.1093/cvr/cvac086

**Published:** 2022-06-11

**Authors:** Coraline Heron, Anais Dumesnil, Mahmoud Houssari, Sylvanie Renet, Theo Lemarcis, Alexis Lebon, David Godefroy, Damien Schapman, Orianne Henri, Gaetan Riou, Lionel Nicol, Jean-Paul Henry, Manon Valet, Marie Pieronne-Deperrois, Antoine Ouvrard-Pascaud, Réné Hagerling, Hélène Chiavelli, Jean-Baptiste Michel, Paul Mulder, Sylvain Fraineau, Vincent Richard, Virginie Tardif, Ebba Brakenhielm

**Affiliations:** Faculty of Pharmacy and Medicine, Normandy University, UniRouen, Inserm (Institut National de la Santé et de la Recherche Médicale) UMR1096 (EnVI Laboratory), FHU CARNAVAL, Rouen, France; Faculty of Pharmacy and Medicine, Normandy University, UniRouen, Inserm (Institut National de la Santé et de la Recherche Médicale) UMR1096 (EnVI Laboratory), FHU CARNAVAL, Rouen, France; Faculty of Pharmacy and Medicine, Normandy University, UniRouen, Inserm (Institut National de la Santé et de la Recherche Médicale) UMR1096 (EnVI Laboratory), FHU CARNAVAL, Rouen, France; Faculty of Pharmacy and Medicine, Normandy University, UniRouen, Inserm (Institut National de la Santé et de la Recherche Médicale) UMR1096 (EnVI Laboratory), FHU CARNAVAL, Rouen, France; Faculty of Pharmacy and Medicine, Normandy University, UniRouen, Inserm (Institut National de la Santé et de la Recherche Médicale) UMR1096 (EnVI Laboratory), FHU CARNAVAL, Rouen, France; Faculty of Biological Sciences, Normandy University, UniRouen, PRIMACEN, Mont Saint Aignan, France; Faculty of Biological Sciences, Normandy University, UniRouen, Inserm UMR1239 (DC2N Laboratory), Mont Saint Aignan, France; Faculty of Biological Sciences, Normandy University, UniRouen, PRIMACEN, Mont Saint Aignan, France; Faculty of Pharmacy and Medicine, Normandy University, UniRouen, Inserm (Institut National de la Santé et de la Recherche Médicale) UMR1096 (EnVI Laboratory), FHU CARNAVAL, Rouen, France; Faculty of Pharmacy and Medicine, Normandy University, UniRouen, Inserm (Institut National de la Santé et de la Recherche Médicale) UMR1234 (PANTHER Laboratory), Rouen, France; Faculty of Pharmacy and Medicine, Normandy University, UniRouen, Inserm (Institut National de la Santé et de la Recherche Médicale) UMR1096 (EnVI Laboratory), FHU CARNAVAL, Rouen, France; Faculty of Pharmacy and Medicine, Normandy University, UniRouen, Inserm (Institut National de la Santé et de la Recherche Médicale) UMR1096 (EnVI Laboratory), FHU CARNAVAL, Rouen, France; Faculty of Pharmacy and Medicine, Normandy University, UniRouen, Inserm (Institut National de la Santé et de la Recherche Médicale) UMR1096 (EnVI Laboratory), FHU CARNAVAL, Rouen, France; Faculty of Pharmacy and Medicine, Normandy University, UniRouen, Inserm (Institut National de la Santé et de la Recherche Médicale) UMR1096 (EnVI Laboratory), FHU CARNAVAL, Rouen, France; Faculty of Pharmacy and Medicine, Normandy University, UniRouen, Inserm (Institut National de la Santé et de la Recherche Médicale) UMR1096 (EnVI Laboratory), FHU CARNAVAL, Rouen, France; Charité – Universitätsmedizin Berlin, corporate member of Freie Universität Berlin and Humboldt-Universität zu Berlin, Institute of Medical and Human Genetics, Augustenburger Platz 1, 13353 Berlin, Germany; Berlin Institute of Health at Charité – Universitätsmedizin Berlin, BIH Center for Regenerative Therapies, Augustenburger Platz 1, 13353 Berlin, Germany; Faculty of Pharmacy and Medicine, Normandy University, UniRouen, Inserm (Institut National de la Santé et de la Recherche Médicale) UMR1096 (EnVI Laboratory), FHU CARNAVAL, Rouen, France; UMR 1148, Inserm-Paris University, X. Bichat Hospital, Paris, France; Faculty of Pharmacy and Medicine, Normandy University, UniRouen, Inserm (Institut National de la Santé et de la Recherche Médicale) UMR1096 (EnVI Laboratory), FHU CARNAVAL, Rouen, France; Faculty of Pharmacy and Medicine, Normandy University, UniRouen, Inserm (Institut National de la Santé et de la Recherche Médicale) UMR1096 (EnVI Laboratory), FHU CARNAVAL, Rouen, France; Faculty of Pharmacy and Medicine, Normandy University, UniRouen, Inserm (Institut National de la Santé et de la Recherche Médicale) UMR1096 (EnVI Laboratory), FHU CARNAVAL, Rouen, France; Faculty of Pharmacy and Medicine, Normandy University, UniRouen, Inserm (Institut National de la Santé et de la Recherche Médicale) UMR1096 (EnVI Laboratory), FHU CARNAVAL, Rouen, France; Faculty of Pharmacy and Medicine, Normandy University, UniRouen, Inserm (Institut National de la Santé et de la Recherche Médicale) UMR1096 (EnVI Laboratory), FHU CARNAVAL, Rouen, France

**Keywords:** Hypertrophy, Vegfc, Vegfd, Inflammation, Wall stress, CCL21, mF4-31C1

## Abstract

**Aims:**

Lymphatics are essential for cardiac health, and insufficient lymphatic expansion (lymphangiogenesis) contributes to development of heart failure (HF) after myocardial infarction. However, the regulation and impact of lymphangiogenesis in non-ischaemic cardiomyopathy following pressure-overload remains to be determined. Here, we investigated cardiac lymphangiogenesis following transversal aortic constriction (TAC) in C57Bl/6 and Balb/c mice, and in end-stage HF patients.

**Methods and results:**

Cardiac function was evaluated by echocardiography, and cardiac hypertrophy, lymphatics, inflammation, oedema, and fibrosis by immunohistochemistry, flow cytometry, microgravimetry, and gene expression analysis. Treatment with neutralizing anti-VEGFR3 antibodies was applied to inhibit cardiac lymphangiogenesis in mice. We found that VEGFR3-signalling was essential to prevent cardiac lymphatic rarefaction after TAC in C57Bl/6 mice. While anti-VEGFR3-induced lymphatic rarefaction did not significantly aggravate myocardial oedema post-TAC, cardiac immune cell levels were increased, notably myeloid cells at 3 weeks and T lymphocytes at 8 weeks. Moreover, whereas inhibition of lymphangiogenesis did not aggravate interstitial fibrosis, it increased perivascular fibrosis and accelerated development of left ventricular (LV) dilation and dysfunction. In clinical HF samples, cardiac lymphatic density tended to increase, although lymphatic sizes decreased, notably in patients with dilated cardiomyopathy. Similarly, comparing C57Bl/6 and Balb/c mice, lymphatic remodelling post-TAC was linked to LV dilation rather than to hypertrophy. The striking lymphangiogenesis in Balb/c was associated with reduced cardiac levels of macrophages, B cells, and perivascular fibrosis at 8 weeks post-TAC, as compared with C57Bl/6 mice that displayed weak lymphangiogenesis. Surprisingly, however, it did not suffice to resolve myocardial oedema, nor prevent HF development.

**Conclusions:**

We demonstrate for the first time that endogenous lymphangiogenesis limits TAC-induced cardiac inflammation and perivascular fibrosis, delaying HF development in C57Bl/6 but not in Balb/c mice. While the functional impact of lymphatic remodelling remains to be determined in HF patients, our findings suggest that under settings of pressure-overload poor cardiac lymphangiogenesis may accelerate HF development.

## Graphical Abstract

**Graphical Abstract:**
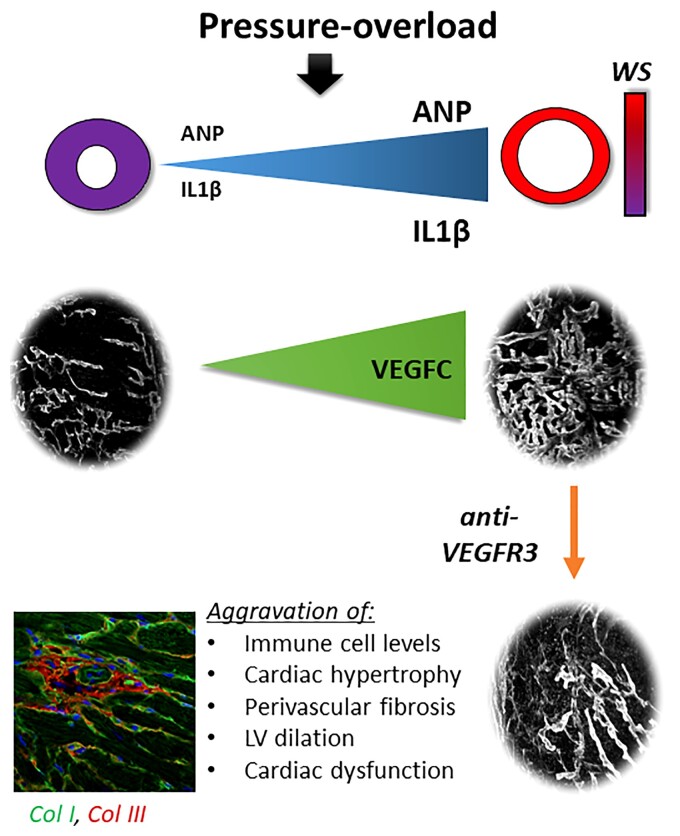
Pressure-overload in mice leads to strain-dependent cardiac responses, with the development of either compensated or decompensated hypertrophy, accompanied by left ventricular (LV) dilation causing increased ventricular wall stress (WS). The compensated hypertrophic phenotype post-transversal aortic constriction (post-TAC), seen in female C57Bl/6 mice, was associated with transient and weak cardiac lymphangiogenesis, accumulation of immune cells and perivascular fibrosis. In contrast, both male C57Bl/6 and female Balb/c mice developed a dilated phenotype post-TAC, characterized by increased cardiac gene expression of ANP and IL1β, and stimulation of cardiac lymphangiogenesis. Inhibition of lymphangiogenesis during pressure-overload uncovered lymphatic rarefaction in the heart, with increased cardiac pro-inflammatory immune cell levels and aggravation of perivascular fibrosis, leading to accelerated development of cardiac remodelling and decompensation.

## 1. Introduction

Cardiac lymphatics are essential for maintenance of cardiac health by ensuring tissue fluid homeostasis through the absorption and return of excess extracellular fluid and solutes, and metabolic waste products, from the heart back to the blood circulation.^1^ In addition, the cardiac lymphatic vasculature, similar as in other organs, regulates immune responses to injury. Indeed, cardiac lymphatics have the potential to modulate immunity both locally in the myocardium, by actively recruiting and evacuating cardiac-infiltrating immune cells, and distally, at the level of cardiac-draining lymph nodes (dLNs), by mediating the uptake from the myocardial interstitium of cytokines, auto-antigens, or pathogens for delivery to dLNs. In addition, recent research has revealed that lymphatic endothelial cells may act directly as antigen-presenting cells, potentially favouring tolerance to self.^2^

Cardiac inflammation is a current therapeutic target in cardiovascular diseases, notably acute and chronic heart failure (HF). Whereas the contribution of innate immune cells to cardiovascular remodelling, including after myocardial infarction (MI), has been increasingly recognized,^3^ the role of adaptive immunity, both B and T lymphocytes, in HF development has been less investigated. Intriguingly, recent experimental and clinical findings indicate that MI and HF are associated with signs of auto-immune disease, including development of autoreactive T cells and anti-cardiac auto-antibodies.^4,5^

In this context, therapeutic lymphangiogenesis emerges as a novel modality to limit both chronic oedema and inflammation in the heart post-MI to prevent HF development.^6–8^ Recently, experimental findings on the beneficial impact of lymphatics during cardiac remodelling have been extended to models of non-ischaemic hypertensive heart disease, induced by either salt-loading in rats or chronic angiotensin-II infusion in mice.^9,10^ Promisingly, lymphangiogenic therapy with Vegfc limited cardiac hypertrophy in these settings. However, as the treatment also prevented kidney dysfunction and reduced chronic hypertension,^10,11^ it still is unknown how much of the functional cardiac benefit observed was directly due to expansion of lymphatics in the heart. In particular, the question remains whether insufficient cardiac lymphangiogenesis may contribute to the chronic myocardial oedema, inflammation, fibrosis, and cardiac remodelling that occurs during pathological cardiac hypertrophy in response to pressure-overload, such as in patients with aortic stenosis.

We hypothesized that increased cardiac wall stress during pressure-overload may induce Vegfc and/or Vegfd growth factors to stimulate cardiac lymphangiogenesis, as previously reported for Vegfa and cardiac angiogenesis during physiological cardiac growth.^12,13^ Furthermore, we postulated that insufficient lymphangiogenesis during cardiac hypertrophy may accelerate HF development and cardiac decompensation [reduced cardiac output (CO)], similar as previously demonstrated for insufficient angiogenesis.^14^ Here, we examined the regulation and functional impact of lymphangiogenesis in the heart during the switch from compensated to decompensated cardiac hypertrophy using mouse models of pressure-overload induced by transversal aortic constriction (TAC). In particular, we took advantage of the differing cardiac responses to pressure-overload previously reported in C57Bl/6 and Balb/c mouse strains,^15^ to compare the regulation of lymphangiogenesis during hypertrophic vs. more dilated cardiac remodelling. Furthermore, we examined lymphatic remodelling in patients with end-stage HF of either ischaemic or non-ischaemic origin, including hypertrophic cardiomyopathy (HCM) and dilated cardiomyopathy (DCM).

## 2. Methods

Cardiac lymphatic remodelling was investigated in adult male and female C57Bl/6 mice and female Balb/c mice (Janvier Laboratories, France) following TAC. Briefly, a minimally invasive method^16^ was used to constrict the aortic arch, using a 26G needle. Double-banding of the aorta was applied to prevent suture internalization and model variability, as described.^17^ Modulation of cardiac lymphangiogenesis was performed using a rat anti-mouse VEGFR3-blocking antibody, mF4-31C1 (Imclone/Eli Lilly), as described.^18^ The antibody therapy consisted of repeated (starting from Day 7 post-TAC) twice a week i.p. injections of 800 µg/mouse of mF4-31C1 or rat IgG as a control. Cardiac samples from end-stage HF patients (recipients of cardiac transplants at the Bichat Hospital in Paris, France), obtained after informed consent, were examined by immunohistology. Discarded cardiac autopsy samples, obtained from donors without cardiovascular disease, were used as healthy controls. For details see *Supplementary Methods*.

### 2.1 Anaesthetics and analgesia

Mice were anaesthetized before TAC surgery by intraperitoneal injection of ketamine (100 mg kg^−1^ Imalgene®) and xylazine (10 mg kg^−1^ Rompun® 2%, Bayer Health Care). Buprenorphine (50 µg kg^−1^, Buprecare®, Axcience) was injected subcutaneously 6 h after surgery and twice per day until 3 days post-operation. For transthoracic echocardiography animals were anaesthetized with isoflurane (1–2%). At the end of the study, animals were euthanized by barbiturate over-dose (100 mg kg^−1^ intraperitoneal, Sodium Thiopental, Rotexmedica).

### 2.2 Cardiac functional, cellular, and molecular analyses in mice

Cardiac function was evaluated by echocardiography.^8^ Cardiac sections were analysed by histology and immunohistochemistry to determine lymphatic and blood vessel densities and sizes, immune cell infiltration, cardiomyocyte hypertrophy, and fibrosis. Whole-mount staining of cardiac lymphatics and arterioles was performed, using a modified iDISCO+ clearing protocol, for imaging by light sheet (ultramicroscope II, LaVision BioTec) and confocal laser scanning (Leica SP8) microscopy.^8^ Cardiac gene expression was analysed by qPCR.^8^ For details, see *Supplementary Methods*.

### 2.3 Flow cytometry

Cardiac immune cells were analysed by flow cytometry using a LSR Fortessa (BD Biosciences) cytometer.^8^ Results are expressed as cells per mg cardiac tissue. For details, see *Supplementary Methods*.

### 2.4 Study approval

Anonymized human heart samples were obtained, following informed consent, by the Bichat hospital biobank (*U1148 BB-0033-00029/*BBMRI, coordinator JB Michel) authorized for tissue collection by the Inserm institutional review board and conforming to the principles outlined in the Declaration of Helsinki. Animal experiments were approved by the regional Normandy ethics review board in line with E.U. and French legislation (01181.01/APAFIS #8157-2016121311094625 v5 and APAFIS #23175-2019112214599474 v6). A total of 243 C57Bl/6 male or female mice and 82 Balb/c female mice, surviving TAC or sham-operation were included in this study.

### 2.5 Statistics

Data are presented as mean ± S.E.M. Comparisons were selected to determine: (1) impact of pathology (healthy sham vs. TAC for each strain and gender) and (2) effect of treatment (anti-VEGFR3-treated vs. IgG TAC controls). Statistical analyses for comparisons of two independent groups were performed using either Student’s two-tailed *t*-test for groups with normal distribution, or alternatively by Mann–Whitney *U* test for samples where normality could not be ascertained based on D’Agostino & Pearson omnibus normality test. For comparisons of three groups or more either one-way analysis of variance (ANOVA) followed by Bonferroni *post hoc* (for parameters with *n* > 7 with normal distribution), or alternatively Kruskal–Wallis non-parametric analysis followed by Dunn’s *post hoc* multiple comparison (for parameters with non-Gaussian distribution) were performed. Longitudinal echocardiography studies were analysed by paired two-way ANOVA followed by Bonferroni *post hoc*, while morphometric data and gene expression data were analysed by two-way ANOVA followed by Sidak’s *post hoc* for pair-wise comparisons or Dunnett’s *post hoc* to compare three or more groups. Non-parametric Spearman rank order tests were used for evaluating correlations. All analyses were performed using GraphPad Prism software.

## 3. Results

### 3.1 Strain- and gender-dependent cardiac remodelling following pressure-overload

**Figure 1:**
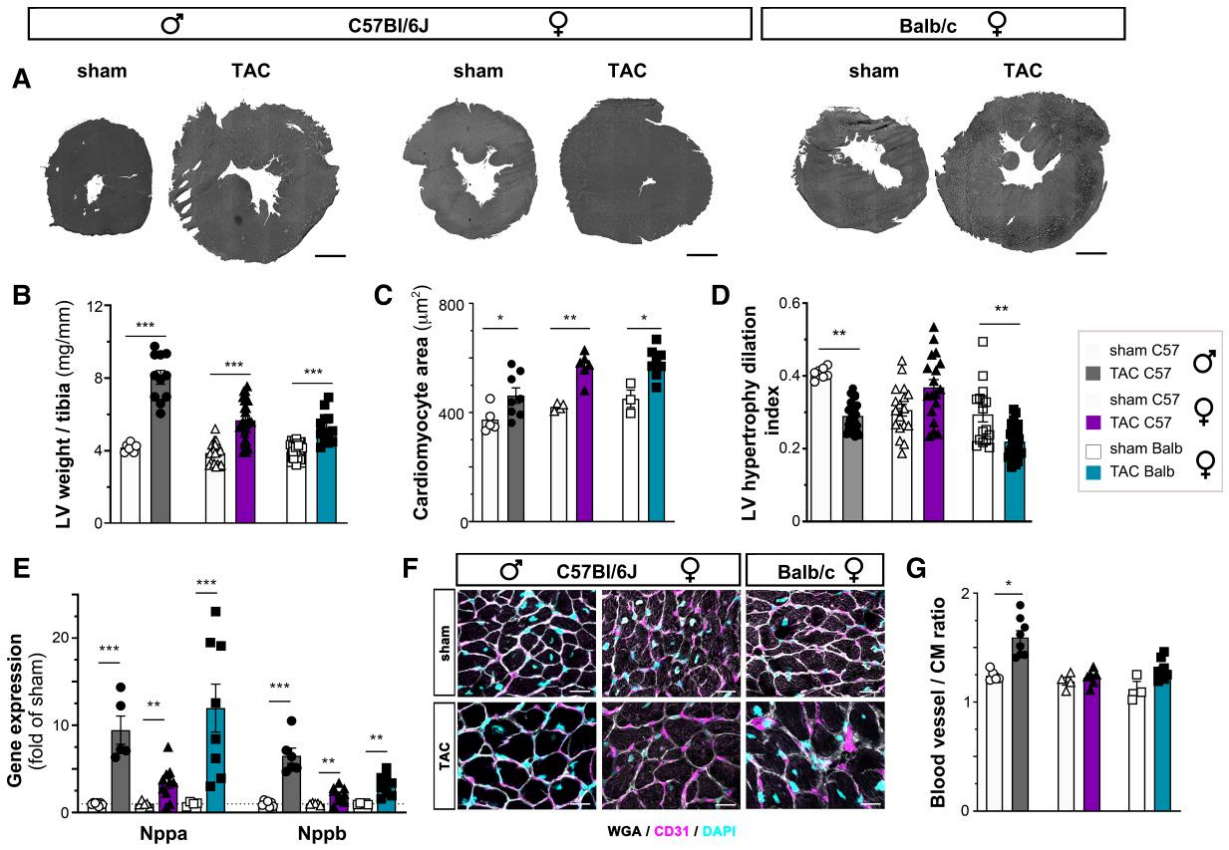
Evaluation of cardiac hypertrophy and remodelling at 8 weeks post-TAC. Examples (*A*, scale bar 1 mm) and morphometric assessment of LV weights normalized to tibia lengths (*B*) at 8 weeks in male C57Bl/6 mice: sham (open circles, *n* = 13) or TAC (closed circles, *n* = 15); female C57Bl/6 mice: sham (open triangles, *n* = 14) or TAC (closed triangles, *n* = 10); and in Balb/c female mice: sham (open square, *n* = 14) or TAC (closed square, *n = 8*). Analysis of cardiomyocyte cross-sectional area (*C*, *n* = 3–8 animals per group) and calculation of LV hypertrophy dilatation index (*D*) at 8 weeks. Cardiac expression analyses at 8 weeks of *Nppa* and *Nppb* (*E*, *n* = 5–10 per group). Examples (*F*) and evaluation at 8 weeks post-TAC of blood vessel to cardiomyocyte ratios (*G*, *n* = 3–8 animals per group). WGA, *white*, CD31, *purple*, Dapi, *blue* (scale bar 20 µm). Groups were compared pair-wise by two-way ANOVA followed by Sidak’s multiple comparison test (for morphometry and cardiac expression analyses) or by non-parametric Kruskal Wallis followed by Dunn’s *post hoc* test (for immunohistochemistry) *P < 0.05, **P < 0.01, ***P < 0.001 vs. sham.

TAC rapidly led to severe cardiac hypertrophy in C57Bl/6 mice, with a 50% increase in left ventricular (LV) mass observed by 3 weeks (see Supplementary material online, *Figure S1a*). At 8 weeks post-TAC, the cardiac hypertrophy in C57Bl/6 mice had stabilized, with a 30% increase in cardiomyocyte sizes as compared with sham-operated controls (*Figure 1a–c*). In contrast, Balb/c mice displayed a weaker initial hypertrophic response, despite a similar increase post-TAC in transaortic pressure gradients in both strains, and only by 8 weeks was the LV mass and cardiomyocyte hypertrophy significantly increased (*Figure 1a–c*; see Supplementary material online, *Figure S1a*). Whereas the pressure-overload led to compensated cardiac hypertrophy (increased LV wall thickness, no LV dilation, and preserved cardiac function) in female C57Bl/6, the other TAC groups displayed little or no increase in LV wall thickness, significantly increased LV chamber dimensions, and cardiac decompensation (see Supplementary material online, *Tables S1–S3*). Indeed, whereas the LV hypertrophy/dilatation index (LV systolic wall thickness/LV EDD) was slightly increased post-TAC in C57Bl/6 females, it significantly decreased in the other TAC groups, illustrating the divergence in cardiac remodelling responses to pressure-overload, notably between female C57Bl/6 and Balb/c mice (*Figure 1d*). Moreover, while body weight gain was unaffected by TAC in C57Bl/6, it was slightly reduced by 8 weeks in Balb/c (see Supplementary material online, *Figure S1b*). In line with earlier onset of HF, as evidenced by reduced CO, in male C57Bl/6 and female Balb/c TAC mice, as compared with female C57Bl/6 (see Supplementary material online, *Tables S1–S3*), analyses of cardiac expression of ANP and BNP genes (*Nppa* and *Nppb*, respectively), used as indicators of cardiac wall stress,^19,20^ similarly revealed an earlier and more robust increase post-TAC in the first two groups (see Supplementary material online, *Figure S1c and e*). The cardiac angiogenic response correlated with the degree of LV dilation (see Supplementary material online, *Figure S1d*), with the largest increase in blood vessel to cardiomyocyte ratios at 8 weeks post-TAC observed in male C57Bl/6, with a smaller increase in female Balb/c, and no change in female C57Bl/6 mice (*Figure 1f and g*). This is in line with the reported impact of wall stress, linked notably to LV dilation, in induction of Vegfa.^12,13^

### 3.2 Regulation of cardiac lymphangiogenesis following pressure-overload

**Figure 2:**
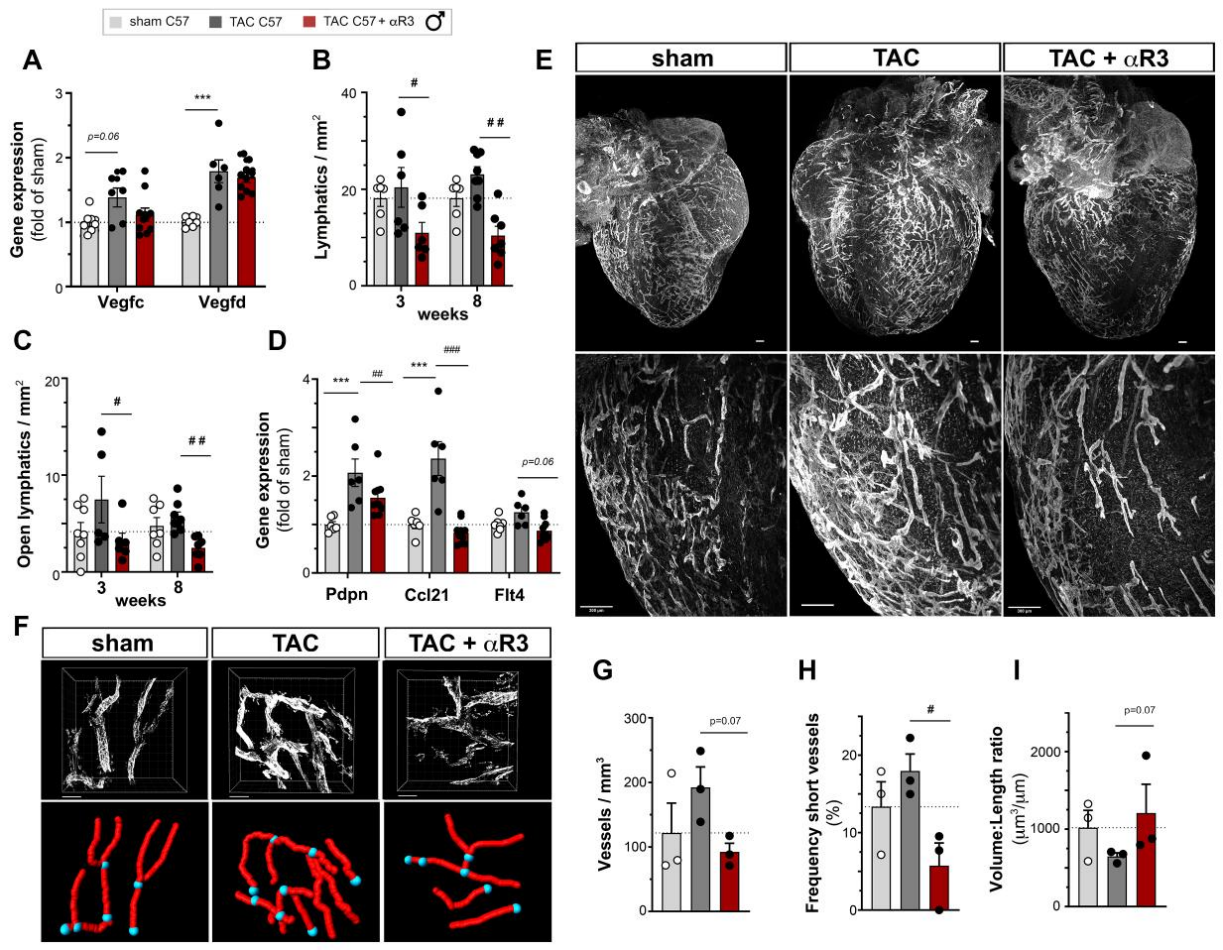
Lymphangiogenesis is necessary to prevent cardiac lymphatic rarefaction post-TAC. Cardiac expression analyses at 8 weeks of *Vegfc* and *Vegfd* (*A*), and evaluation at 3 and 8 weeks of lymphatic vessel density (*B*) and open lymphatic density (*C*) in the LV subepicardium in male C57Bl/6 sham (open circles, *n = 6*), TAC controls (closed circles, *n* = 6–8), and anti-VEGFR3-treated TAC (closed circles, *n* = 6–7). Cardiac expression analyses at 8 weeks of lymphatic markers *Pdpn, Ccl21*, and *Flt4* (*D*, *n* = 6–10 animals per group). Light sheet imaging of cardiac lymphatics (*E*) at 3 weeks post-TAC visualized by Lyve1 staining (scale bar 300 µm). Examples of 3D modelling of cardiac lymphatics visualized by confocal analyses (Lyve1) (*F*, scale bar 100 µm). Quantification of cardiac lymphatic volume density (*G*), frequency of short lymphatic branches (*H*), and volume:length ratio (*I*) (*n = 3* mice per group). Groups were compared by non-parametric Kruskal Wallis followed by Dunn’s *post hoc* test (for immunohistochemistry) and by two-way ANOVA followed by Dunnett’s multiple comparisons test (for expression analyses). *P < 0.05 vs. sham, ##*P* < 0.01, ###*P* < 0.001 vs. TAC control.

Next, we investigated cardiac lymphatic remodelling, hypothesizing that cardiac lymphangiogenesis would occur post-TAC to match the drainage capacity to the expanded LV mass. However, both cardiac gene expression analyses and immunohistochemistry revealed that at 3 weeks post-TAC there was only a slight, non-significant increase in cardiac *Vegfc* and *Vegfd* levels (see Supplementary material online, *Figure S2a*), and in lymphatic density (*Figure 2b*), in C57Bl/6 males, despite their extensive cardiac hypertrophy. Further, although *Ccl21* chemokine expression was increased (see Supplementary material online, *Figure S2b*), indicating lymphatic activation in the heart, the open lymphatic density was not significantly increased (*Figure 2c*). However, cardiac pre-collector slimming, previously reported post-MI in rodents,^6,8^ did not occur, and subsequently open cardiac lymphatic area remained unchanged (see Supplementary material online, *Figure S2c*). Only by 8 weeks post-TAC was the cardiac expression of *Vegfc*, and especially *Vegfd*, significantly increased (*Figure 2a*). In agreement, the expression of lymphatic markers podoplanin (*Pdpn*) and *Ccl21* were increased (*Figure 2d*), together with slightly increased lymphatic density (*Figure 2b and e*). Thus, the rapid and severe cardiac growth induced by TAC in C57Bl/6 males was associated with a slow and weak lymphangiogenic response.

### 3.3 Inhibition of lymphangiogenesis aggravates cardiac hypertrophy and accelerates cardiac dysfunction

To determine whether such a modest lymphangiogenic response in male C57Bl/6 mice may still have protected against cardiac inflammation and/or decompensation after pressure-overload, we next investigated the impact of selective inhibition of lymphangiogenesis using a VEGFR3-blocking antibody, mF4-31C1.^18^ To not interfere with the initial response to the pressure-overload, administration of the blocking antibody, or non-specific rat IgG in TAC controls, was initiated at 1 week after surgery, and the treatment was then maintained throughout the 8-week study. We found that the anti-VEGFR3 treatment rapidly, selectively, and completely blocked cardiac lymphangiogenesis post-TAC, despite persistently elevated cardiac *Vegfd* expression (*Figure 2a–d*; see Supplementary material online, *Figure S2a–d*). Indeed, while the treatment prevented upregulation of cardiac *Ccl21* expression already at 3 weeks post-TAC (see Supplementary material online, *Figure S2b*), by 8 weeks all investigated lymphatic markers (*Pdpn, Ccl21, Flt4*) were reduced (*Figure 2d*). This reveals that the endogenous lymphangiogenic response post-TAC is essential not to expand, but to maintain lymphatic density in the hypertrophic heart. Indeed, prevention of lymphangiogenesis led to rarefaction of lymphatic capillaries and pre-collectors, as demonstrated by cardiac whole-mount imaging (*Figure 2e*). Three-dimensional analyses further revealed a reduction of both lymphatic volume density and the frequency of short-vessel segments, indicating pruning of lymphatic sprouts post-TAC in the setting of reduced VEGFR3 signalling in anti-VEGFR3-treated mice (*Figure 2f–i*). In contrast, the treatment had no effect on cardiac angiogenesis or arteriogenesis, nor on cardiac perfusion, as evaluated by MRI (see Supplementary material online, *Figure S3*).

**Figure 3:**
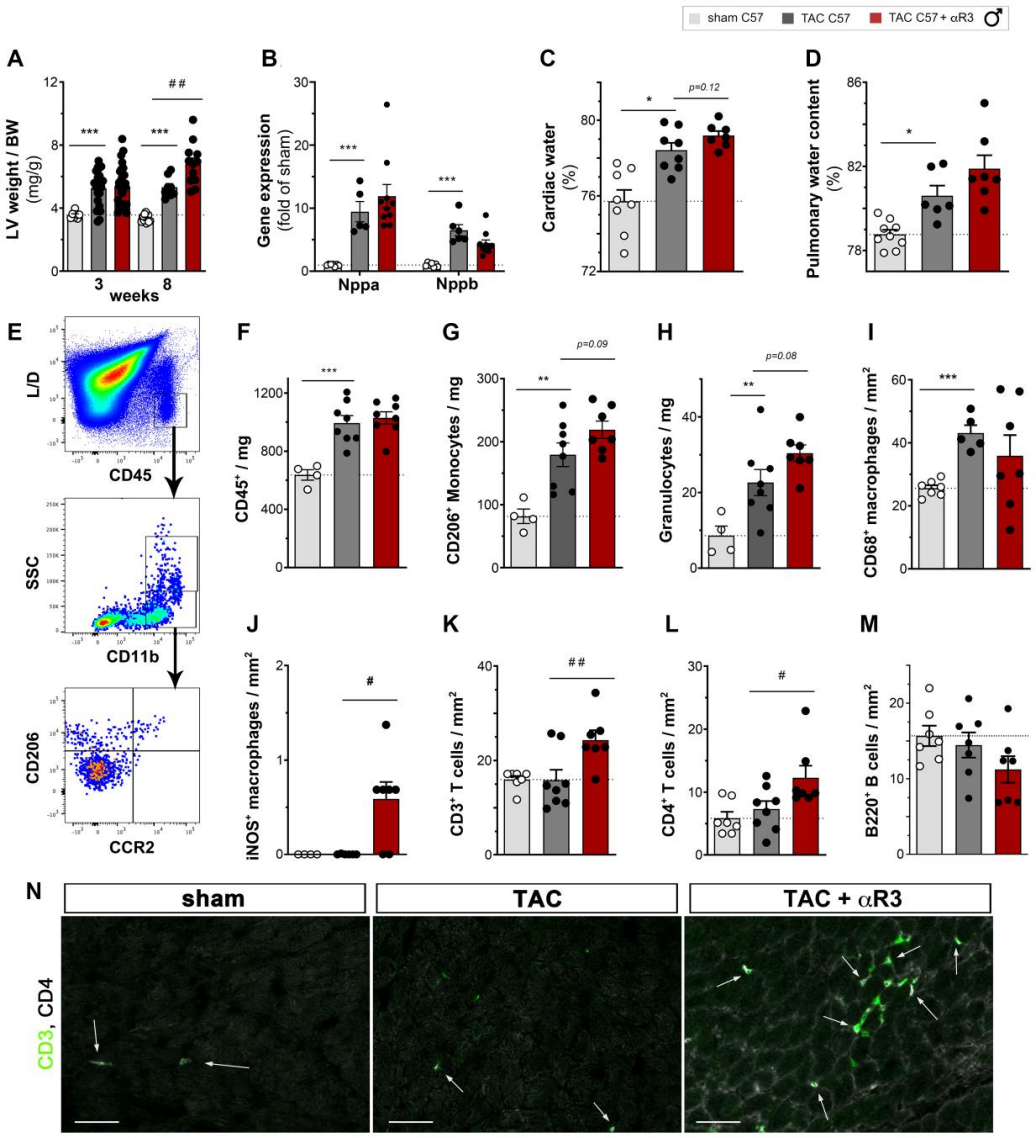
Aggravation of cardiac hypertrophy and inflammation, but not oedema, by anti-VEGFR3 treatment. Morphometric assessment of LV weight normalized to body weight (*A*) at 3 or 8 weeks in male C57Bl/6 sham (open circles, *n* = 7–13), TAC controls (closed circles, *n* = 8–20); and anti-VEGFR3-treated TAC (closed circles, *n* = 12–20). Analysis of cardiac *Nppa* and *Nppb* (*B*) expression at 8 weeks (*n* = 6–10 per group). Assessment of cardiac (*C*) and pulmonary (*D*) water content at 3 weeks post-TAC in male C57Bl/6 (*n* = 6–9 per group). Flow cytometric evaluation (*E*) at 3 weeks post-TAC (*n* = 4–8 per group) of cardiac-infiltrating CD45^+^ immune cells (*F*), CD11b^+^ CD206^+^ monocytes (*G*) and CD11b^+^ SSC^high^ granulocytes (*H*). Data are reported as *cells per mg* cardiac tissue. Quantification by immunohistochemistry at 3 weeks post-TAC (*n* = 6–7 animals per group) of total cardiac-infiltrating CD68^+^ macrophages (*I*), and classical iNOS^+^ macrophages (*J*). Quantification by immunohistochemistry at 8 weeks post-TAC (*n* = 7–8 animals per group) of total cardiac-infiltrating CD3^+^ T cells (k), CD4^+^ T cells (*L*), and B220^+^ B cells (*M*). Examples of cardiac CD3^+^ total T cells (g*reen*) and CD4^+^ T cell subpopulation (*grey*) indicated by arrows (*N*). Scalebar 50 µm. Groups were compared by non-parametric Kruskal Wallis followed by Dunn’s *post hoc* test (for immunohistochemistry, microgravimetry, and flow cytometry) and by two-way ANOVA followed by Sidak’s multiple comparison test (for morphometric data) or Dunnett’s multiple comparisons test (for expression analyses). *P < 0.05, **P < 0.01, ***P < 0.001 vs. sham, ##*P* < 0.01, ###*P* < 0.001 vs. control TAC.

Concerning the cardiac hypertrophic response to pressure-overload, there was a more pronounced increase in LV mass in the anti-VEGFR3-treated group by 8 weeks (*Figure 3a*). In contrast, there was no change in body weight gain (see Supplementary material online, *Figure S4a*), and cardiac expression of *Nppa* and *Nppb*, used as biomarkers of wall stress, were similarly increased at 3- and 8-weeks post-TAC in both groups (*Figure 3b*, see Supplementary material online, *Figure S4b*). However, in the anti-VEGFR3-treated group, the development of LV dilation was accelerated, occurring already by 3 weeks post-TAC, as compared with at 6 weeks in controls (see Supplementary material online, *Table S4*). Nevertheless, at the end of the study, the cardiac dysfunction was similar between the two TAC groups, indicating that inhibition of lymphangiogenesis accelerated the progression, but not the severity, of the cardiac decompensation process.

### 3.4 Impact of inhibition of lymphangiogenesis on oedema and resolution of cardiac inflammation

To investigate by which mechanism lymphatic rarefaction may have accelerated cardiac hypertrophy and LV dilation, we analysed cardiac and pulmonary water content at 3 weeks post-TAC. Using microgravimetry, we found that cardiac oedema only tended to increase in the anti-VEGFR3-treated group as compared with TAC controls (*Figure 3c*). In line with the aggravation of cardiac dysfunction by anti-VEGFR3 treatment, pulmonary oedema also tended to increase (*Figure 3d*). Another aspect of lymphatic function is the clearance of immune cells to resolve inflammation. We expected that lymphatic rarefaction in anti-VEGFR3-treated TAC mice may increase immune cell levels in the heart. While pro-inflammatory cytokine and chemokine expression (*Il1b*, *Il6,* and *Ccl2*) were similarly increased in both TAC groups (see Supplementary material online, *Figure S4c*), cardiac flow cytometry at 3 weeks revealed slightly increased granulocyte and monocyte levels in anti-VEGFR3-treated mice (*Figure 3e–h*, see Supplementary material online, *Figure S4d*). In contrast, cardiac T and B cell levels were unchanged as compared with TAC controls (see Supplementary material online, *Figure S4e–i*). Using immunohistochemistry, we further found that the cardiac density of classical pro-inflammatory iNOS^+^ CD206^-^ macrophages, but not total macrophage levels, were strikingly increased in anti-VEGFR3-treated mice (*Figure 3i and j*). Moreover, by 8 weeks, anti-VEGFR3-treated mice displayed significantly increased levels of T cells, but not B cells (*Figure 3k–n*). Notably, CD4^+^ helper T cells, previously demonstrated to accelerate cardiac decompensation,^21^ were increased. In contrast, there was no change in cardiac T regulatory cells (see Supplementary material online, *Figure S4g*). In conclusion, we found that during pressure-overload, the increase in cardiac Vegfc and Vegfd is essential to maintain lymphatic density in the hypertrophic heart. In the absence of VEGFR3 signalling, the clearance of myocardial oedema and especially inflammatory cells is delayed, contributing to accelerated LV remodelling and HF progression.

### 3.5 Regulation of cardiac lymphangiogenesis in compensated vs. decompensated cardiac hypertrophy following pressure-overload

Next, to investigate whether LV dilation, rather than hypertrophy, may drive cardiac lymphangiogenesis, we took advantage of the strain-dependent differences to pressure-overload in C57Bl/6 females as compared with Balb/c females (*Figure 1*). If LV dilation was linked to cardiac lymphatic activation, then more robust lymphangiogenesis would be expected in Balb/c mice.

**Figure 4:**
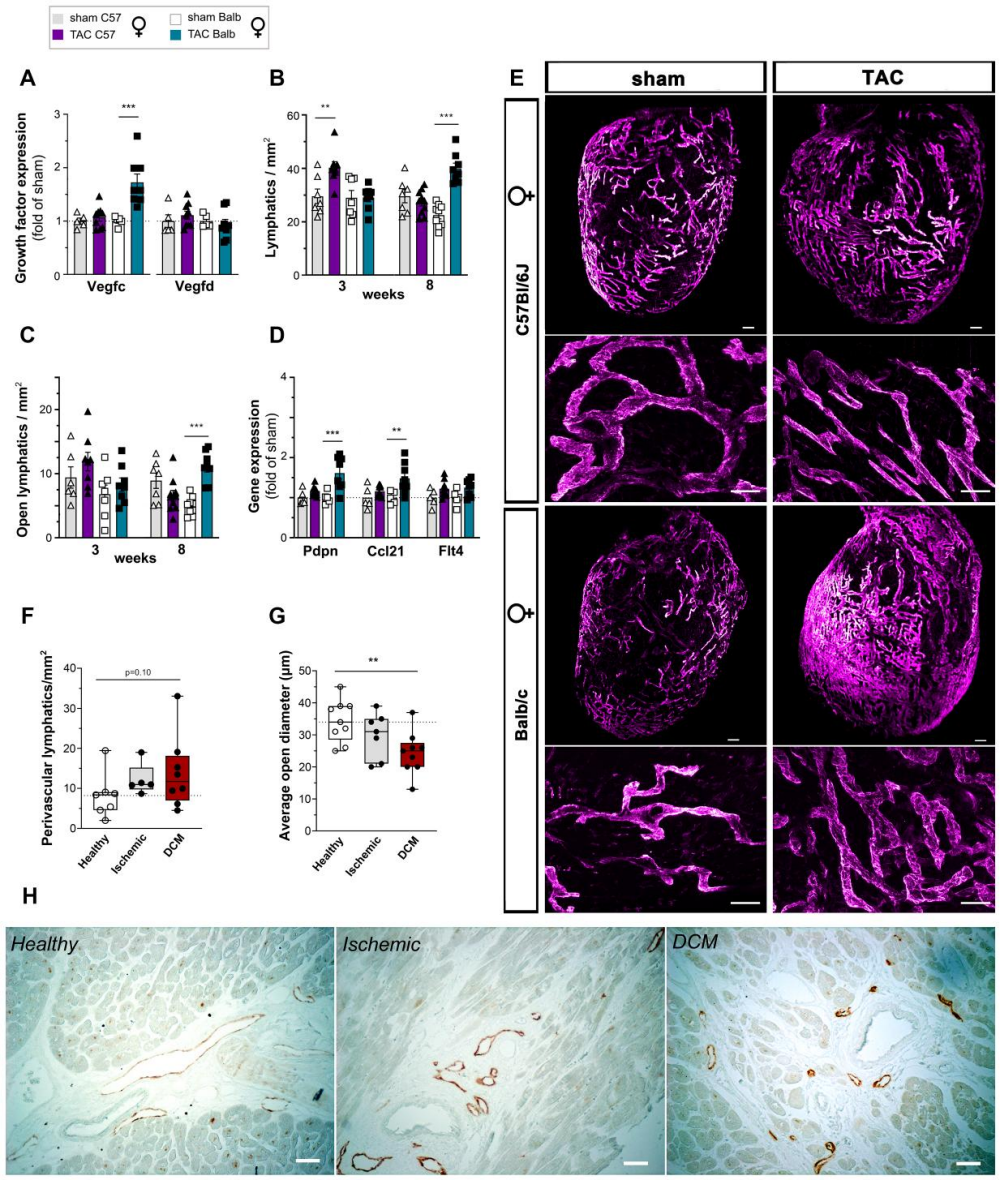
Evaluation of strain-dependent cardiac lymphatic remodelling in mice post-TAC and in HF patients. Cardiac expression analyses (*n* = 5–10 per group) at 8 weeks post-TAC of *Vegfc* and *Vegfd* (*A*) in female C57Bl/6 sham (open triangles) or TAC (closed triangles); and in female Balb/c sham (open square, *n = 9*) or TAC (closed square). Evaluation of total lymphatic density (*B*) and open lymphatic density (*C*) in the LV subepicardium at 3 or 8 weeks (*n* = 7–10 per group). Expression analyses of lymphatic markers *Pdpn, Ccl21*, and *Flt4* (*D*, *n* = 5–10 per group). Examples by light sheet and confocal imaging of cardiac lymphatics (*E*) at 8 weeks post-TAC. Lyve1 (scale bar *upper row* 300 µm, *lower row* zoomed confocal views 100 µm). Quantification of perivascular lymphatic density (*F*), lymphatic lumen sizes (*G*), and examples of cardiac lymphatic density (*H*) in healthy cardiac donors vs. end-stage HF patients with ischaemic or DCM. Podoplanin (scale bar 50 µm). Groups were compared pair-wise by non-parametric Kruskal Wallis followed by Dunn’s *post hoc* test (for immunohistochemistry) or Sidak’s multiple comparisons test (for cardiac expression analyses) *P < 0.05, **P < 0.01, ***P < 0.001 vs. healthy/sham.

In C57Bl/6 females, the compensated cardiac hypertrophy was associated with a transient increase, at 3 weeks post-TAC, of cardiac *Vegfd* expression (see Supplementary material online, *Figure S5a*). Consequently, gene expression of lymphatic markers *Pdpn* and *Ccl21* (see Supplementary material online, *Figure S5b*), cardiac lymphatic density (*Figure 4b and c*), and open lymphatic area (see Supplementary material online, *Figure S5c and d*) were all increased at 3 weeks. However, by 8 weeks, lymphatic growth factor expression, lymphatic density, and gene expression of lymphatic markers had essentially reverted back to initial levels despite the pronounced hypertrophy (*Figure 4a–e*).

In Balb/c females, where the same pressure-overload induced a slower hypertrophic response, there was no cardiac lymphangiogenesis at 3 weeks post-TAC (*Figure 4b and c*; see Supplementary material online, *Figure S5*). However, the dilated cardiac phenotype developing at 6–8 weeks in female Balb/c mice (see Supplementary material online, *Table S2*), indicative of severe and chronically-elevated cardiac wall stress, was associated with a strong and selective increase in cardiac *Vegfc* gene expression (*Figure 4a*). This led to a remarkably potent lymphangiogenic response by 8 weeks (*Figure 4b–d*), with expansion mostly of lymphatic capillaries, as observed by whole-mount imaging (*Figure 4e*). Interestingly, following pressure-overload, both *Vegfc* and *Vegfd* cardiac gene expression positively correlated with augmented wall stress, approximated by increased cardiac *Nppa* and *Nppb* levels (see Supplementary material online, *Figure 6a and b*), rather than with the degree of cardiac hypertrophy (increased LV mass).

In humans, we found that the perivascular lymphatic density in the heart was increased in HF patients with primary DCM (*Figure 4f and h*) and HCM (*Table 1*), as compared with healthy controls. However, lymphatic sizes were significantly reduced (*Figure 4g and h*), notably in DCM patients, suggesting that lymphatic transport capacity may be limited.

**Table 1:** Immunohistochemical analyses of cardiac lymphatics in HF patients and healthy controls

	*n*	Age (years)	Male gender (%)	Open lymphatics/mm^2^	Lymphatic diameter (µm)	Perivascular lymphatics/mm^2^
Healthy controls	9	**60** ± 5	89	**9** ± 2	**34** ± 2	**8** ± 2
ICM	7	**54** ± 5	86	**9** ± 2	**30** ± 3	**12** ± 2
Primary DCM	9	**49** ± 4	78	**9** ± 2	**24** ± 2*	**14** ± 3
HCM	4	**61** ± 7	100	**10** ± 3	**26** ± 3	**17** ± 4**

Bold indicates mean values.

ICM, ischaemic cardiomyopathy; DCM, dilated cardiomyopathy; HCM, hypertrophic cardiomyopathy.

*P* < 0.01 vs. healthy controls (non-parametric Kruskal–Wallis)

*P* = 0.06.

**Figure 5:**
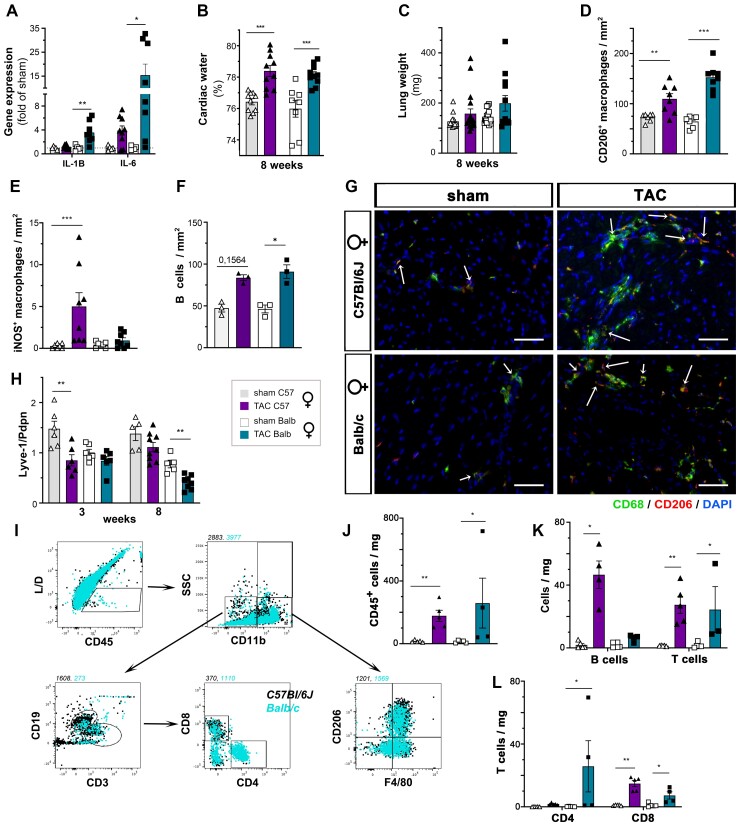
Strain-dependent effects on cardiac inflammation, but not oedema, during pressure-overload. Cardiac expression analysis of *Il1b* and *Il6* (*A*) at 8 weeks post-TAC (*n* = 5–10 per group) in female C57Bl/6 sham (open triangles) or TAC (closed triangles), and in female Balb/c sham (open square) or TAC (closed square). Assessment of cardiac water content (*B*), and pulmonary weights (*C*) at 8 weeks (*n* = 8–10 per group). Immunohistochemical analyses, at 3 weeks, of cardiac CD206^+^ macrophages (*D*), classical iNOS^+^ macrophages (*E*), and B220^+^ B cells (*G*) (*n* = 7–8 per group). Examples of cardiac macrophages (*F*). CD68, *green*, CD206, *red*; dapi *blue*. Arrows indicate CD206^+^ macrophages (scalebar, 50 µm). Analysis of cardiac *Lyve-1* expression, normalized to *Pdpn* levels, at 3- or 8-weeks post-TAC (*H*). Examples of flow cytometry gating in TAC mice (*I*). Quantification in C57Bl/6 and Balb/c females at 8 weeks post-TAC (*n* = 4–5 *samples* per group) of cardiac-infiltrating CD45^+^ immune cells (*J*), CD19^+^ B cells *vs* CD3^+^ T cells (*K*), and CD4^+^ vs. CD8^+^ T cell subpopulations (*L*). Data is reported as *cells per mg* cardiac tissue. Groups were compared pair-wise by two-way ANOVA followed by Sidak’s multiple comparison tests (morphometry data and expression analyses) or non-parametric Kruskal Wallis followed Dunn’s *post hoc* test (for microgravimetry and immunohistochemistry) or by Mann–Whitney *U* test (for flow cytometry); *P < 0.05, **P < 0.01, ***P < 0.001 vs. sham.

In our mouse model, the cardiac lymphangiogenic response may be influenced by strain-dependent differences related to the cytokine environment, with significantly higher cardiac expression observed post-TAC in Balb/c mice of the pro-inflammatory cytokines interleukin (IL)-1β and IL-6, as compared with in C57Bl/6 mice (*Figure 5a*). Previous work has shown that both these cytokines induce *Vegfc* production in fibroblasts and macrophages to stimulate lymphangiogenesis in other organs.^22–24^ Indeed, immunohistochemical analyses revealed prominent Vegf-c protein expression by cardiac macrophages, while Vegf-d protein was predominantly detected in myocardial blood vessels, both in healthy mice and after TAC (see Supplementary material online, *Figure S7*). Moreover, we found that cardiac *Il1b* levels, better than *Il6*, positively correlated with *Vegfc*, but not with *Vegfd* or *Vegfa*, gene expression in the heart (see Supplementary material online, *Figure S6c*). Thus, one explanation for the more potent lymphatic response to pressure-overload in Balb/c mice may be the higher cardiac *Il1b* and *Il6* levels that, together with elevated wall stress due to LV dilation, drive *Vegfc* expression in the heart.

### 3.6 Cardiac lymphangiogenesis does not suffice to resolve myocardial oedema after TAC

Next, we set out to investigate the functional effects linked to cardiac lymphatic remodelling in compensated vs. decompensated cardiac hypertrophy. Surprisingly, there was no difference in the degree of cardiac oedema at 8 weeks post-TAC between C57Bl/6 and Balb/c female mice (*Figure 5b*), indicating that the prominent cardiac lymphangiogenesis occurring in the latter was not sufficient to resolve chronic myocardial oedema. In contrast, pulmonary weights only tended to increase at 8 weeks (see Supplementary material online, *Figures S8a and S5c*). Myocardial oedema depends on the balance between blood vascular permeability and lymphatic transport. Thus, the failure of expanded lymphatics to reduce oedema after TAC may reflect either non-functional and leaky lymphatics, or increased blood vascular permeability. Previous studies have demonstrated chronic vascular leakage in the heart after pressure-overload, due to weakening of the blood vascular barrier mediated by TGF-β-induced downregulation of claudin-5 in vascular endothelium.^25^ In agreement, in our study, at 8 weeks post-TAC, there was increased myocardial vascular permeability, notably in Balb/c mice (see Supplementary material online, *Figure S9a*), as determined using immunohistochemical detection of extravasated fibrinogen. In parallel, evaluations of lymphatic barrier properties, by immunohistochemical analyses of button-like junctions in lymphatic capillaries, revealed a reduction in C57Bl/6, but not Balb/c, mice at 8 weeks post-TAC (see Supplementary material online, *Figure 9b and c*). The formation of more continuous, zipper-like junctions in capillaries may prevent or reduce lymphatic uptake of fluids, which, in the setting of increased vascular leakage after TAC, could account for the chronic myocardial oedema observed in C57Bl/6 mice. In contrast, in Balb/c mice, the expanded lymphatic capillaries appeared structurally functional, suggesting that the myocardial oedema resulted from blood vascular leakage overwhelming lymphatic transport capacity in the heart.

### 3.7 Impact of lymphangiogenesis on cardiac immune cell levels

We and others previously demonstrated that therapeutic cardiac lymphangiogenesis limits myocardial inflammation post-MI in rats and mice.^6–8^ Conversely, prior studies have elegantly shown the key role that different immune cells play during the transition from physiological to pathological hypertrophy following pressure-overload.^21,26,27,28^ We thus set out to investigate whether the graded cardiac lymphangiogenic response observed in our TAC models was associated with differential clearance of immune cells. Based on the observed strain-dependent differences in cardiac lymphatic density post-TAC, we expected more efficient resolution of cardiac inflammation in Balb/c vs. in C57Bl/6 female mice.

First, in agreement with our findings in C57Bl/6 male mice, we found, using immunohistochemistry, that the early immune response to pressure-overload in both strains was characterized by increased cardiac myeloid cell levels, notably macrophages (see Supplementary material online, *Figure S8b*), including alternative CD206^+^ macrophages (*Figure 5d and f*, see Supplementary material online, *Table S5*). The level of classical, iNOS^+^ pro-inflammatory macrophages was highest in C57Bl/6 females (*Figure 5e*), which displayed compensated hypertrophy but waning lymphangiogenesis at 3 weeks post-TAC. Further, similar as noted in C57Bl/6 males, cardiac B lymphocyte levels were slightly increased at 3 weeks post-TAC, especially in Balb/c mice (*Figure 5g*).

Beyond lymphatic density, the molecular profile of lymphatic vessels contributes to regulation of immune cell recruitment and uptake.^29^ In agreement with a recently published report,^30^ we observed reduced cardiac lymphatic expression at 3 weeks post-TAC of the hyaluronic acid receptor *Lyve1* in C57Bl/6, but not Balb/c, mice (*Figure 5h*). This may have contributed to limit lymphatic trafficking of hyaluronic acid-coated CD44^+^ immune cells,^29^ despite the transiently expanded lymphatic network in C57Bl/6 mice. In contrast, in Balb/c mice, a reduction in lymphatic *Lyve1* levels only occurred at 8 weeks post-TAC (*Figure 5h*). Further, only in Balb/c mice was there a reduction at 8 weeks post-TAC of lymphatic *Vcam-1*, but not *Icam-1*, expression (see Supplementary material online, *Figure S10*). Thus, although Balb/c mice displayed extensive, but delayed lymphangiogenesis during pressure-overload, the change in molecular profile may lead to inefficient immune cell uptake and transport by the expanded lymphatics.

Indeed, cardiac analyses, at 8 weeks post-TAC, revealed chronically increased immune cell levels in the heart of both C57Bl/6 and Balb/c mice (Figure 5i and j, see Supplementary material online, *Table S5*). This included myeloid cells, both monocytes and macrophages, notably CD206^+^ macrophages in C57Bl/6 mice (see Supplementary material online, *Figure 8c and d*), although levels were reduced as compared with at 3 weeks (see Supplementary material online, *Figure S8b*). Further, while only C57Bl/6 mice displayed increased cardiac B cell and mature natural killer (NK) cell levels (*Figure 5i and k*, see Supplementary material online, *Figure S8e and f*), both TAC groups had increased cardiac T cell levels (*Figure 5k*). Interestingly, while in Balb/c this included expansion of both CD4^+^ and CD8^+^ T cells, in C57Bl/6 mice only CD8^+^ T cells were increased (*Figure 5i and l*). Furthermore, only in C57Bl/6 mice was there an increase in cardiac *Ccl2* and *TNFα* expression at 8 weeks post-TAC (see Supplementary material online, *Figure 8g*), while, as mentioned above, the expression of *Il1b* and *Il6* was elevated in Balb/c mice (*Figure 5a*). However, this significant and chronic cardiac immune response after pressure-overload in C57Bl/6 females was not sufficient to induce overt cardiac dysfunction. Of note, while some studies indicate a key pathological role of B lymphocytes to promote LV dilation in C57Bl/6 males following pressure-overload,^28^ these cells have also been suggested to serve a beneficial role post-TAC by producing immune-suppressive IL10 to signal inflammatory resolution.^27^ These different properties may be carried by distinct B cells subsets, as recently suggested by an elegant cardiac scRNASeq study post-TAC.^31^

Taken together, our data suggest that a poor lymphangiogenic response may contribute to increase cardiac immune cell levels, notably macrophages, NK cells, and B lymphocytes during pathological cardiac hypertrophy. Furthermore, despite extensive lymphangiogenesis, alterations of lymphatic molecular profiles may limit immune cell exit from the heart, notably CD4^+^ T cells, as observed in Balb/c mice post-TAC.

### 3.8 Lymphangiogenesis limits perivascular fibrosis in the heart

Both cardiac oedema and inflammation are potent drivers of cardiac fibrosis.^32^ Thus, we next set out to determine whether poor lymphangiogenesis during pressure-overload, by aggravating myocardial oedema and/or inflammation, may have accentuated cardiac collagen production and/or deposition.

**Figure 6:**
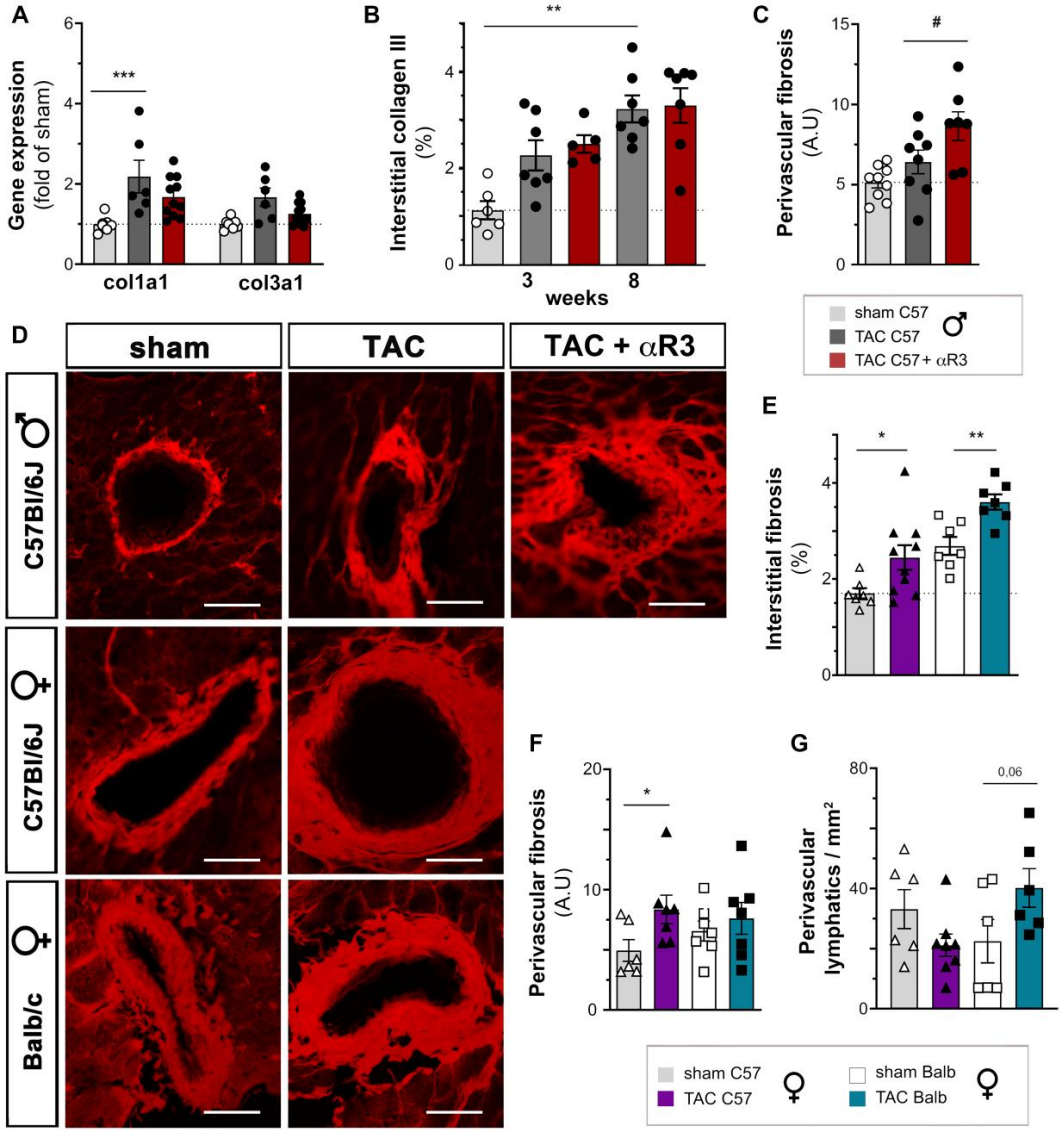
Cardiac interstitial and perivascular fibrosis, and perivascular lymphangiogenesis post-TAC. Cardiac gene expression of *Col1a1* and *Col3a1* (*A*) at 8 weeks post-TAC in male C57Bl/6 sham (open circles), TAC controls (closed circles); and anti-VEGFR3-treated TAC (closed circles) (*n* = 6–10 per group). Quantification of interstitial collagen III density (*B*) at 3 and 8 weeks in male C57Bl/6 mice (*n* = 5–9 per group). Quantification in male C57Bl/6 (*C*) and examples (*D*) of perivascular fibrosis at 8 weeks post-TAC, evaluated as relative fibrotic area surrounding arterioles in the size range of 5–50 µm diameter (scalebar: 50 µm). Quantification of interstitial (*E*) and perivascular (*F*) fibrosis at 8 weeks in female C57Bl/6 sham (open triangles, *n = 7*) or TAC (closed triangles, *n* = 10) and in female Balb/c sham (open square, *n = 7*) or TAC (closed square, *n = 8*). Quantification of perivascular lymphatic density in female C57Bl/6 and Balb/c mice at 8 weeks (*G*, *n* = 6–8 per group). Groups were compared by non-parametric Kruskal Wallis followed by Dunn’s *post hoc* test (for histology) and by two-way ANOVA followed by Dunnett’s multiple comparisons test (for expression analyses). *P < 0.05, **P < 0.01, ***P < 0.001 vs. sham; ##*P* < 0.01, ###*P* < 0.001 vs. control TAC.

First, we investigated cardiac fibrosis in male C57Bl/6 mice treated or not with anti-VEGFR3. Using cardiac gene expression analysis, we found that the levels of collagen 1, but not collagen 3 (*Col1a1* and *Col3a1* genes), were similarly increased at 8 weeks post-TAC in both anti-VEGFR3-treated and control IgG-treated mice, as compared with sham (*Figure 6a*). Interstitial cardiac fibrosis, evaluated by either collagen 1 (see Supplementary material online, *Figure 11a and b*) or collagen 3 (*Figure 6b*) immunohistochemistry, revealed similar increases in both TAC groups at 3 and 8 weeks. In contrast, the level of perivascular fibrosis was significantly increased in anti-VEGFR3-treated mice at 8 weeks post-TAC (*Figure 6c and d*). In support of a local role of lymphatics, light sheet imaging of the region of the septal artery showed expansion of perivascular lymphatics after pressure-overload, which was prevented by anti-VEGFR3 treatment (see Supplementary material online, *Figure S12a*).

Secondly, we examined cardiac fibrosis at 8 weeks post-TAC in C57Bl/6 and Balb/c females using histology. Despite their clear differences in cardiac lymphangiogenesis following pressure-overload, the development of interstitial fibrosis was similar in both strains post-TAC (*Figure 6e*; see Supplementary material online, *Figure 11c*). In contrast, perivascular fibrosis was significantly increased only in C57Bl/6 females, characterized by poor lymphangiogenesis and increased cardiac macrophage, NK and B cell levels (*Figure 6d and f*). In agreement with a local role of lymphatics in prevention of perivascular fibrosis, only in Balb/c mice was there an increase in perivascular lymphatics at 8 weeks post-TAC (*Figure 6g*, see Supplementary material online, *Figure S12b*). Taken together, our data indicate that poor lymphangiogenesis represent as risk factor for perivascular, but not interstitial, fibrosis during pressure-overload, potentially linked to insufficient lymphatic drainage of the immediate surroundings of hypertensive arteries. We thus speculate that the increase in perivascular lymphatic density, seen in clinical HF patients (*Table 1*), may have served to limit perivascular inflammation, oedema and fibrosis.

## 4. Discussion

In this study, we demonstrate that pressure-overload in mice leads to upregulation of lymphangiogenic growth factors, with strain-dependent expansion of cardiac lymphatics linked to LV dilation rather than to LV wall thickening (see Supplementary material online, *Table S6*). Our results are in agreement with previous reports of increased *Vegfc* and *Vegfd* expression during pathological cardiac hypertrophy in mice and men.^30,33–35^ Importantly, inhibition of lymphangiogenesis in mice, by VEGFR3-blocking antibodies, uncovered lymphatic rarefaction post-TAC, which aggravated cardiac inflammation, hypertrophy, and perivascular fibrosis, and accelerated development of cardiac dysfunction and adverse remodelling.

One key to the strain-dependent differences in lymphangiogenesis observed following pressure-overload may be the development of a non-dilated (‘concentric’) cardiac hypertrophic phenotype in C57BL/6 females. This limits wall stress, given that stress correlates positively with LV *pressure* (similar increase post-TAC in both strains) and *chamber size* (larger post-TAC in Balb/c vs female C57Bl/6). Indeed, cardiac stress, estimated by cardiac *Nppa* expression, was considerably lower, by 6–8 weeks post-TAC, in female C57Bl/6 as compared with Balb/c. We postulate that this may have contributed to reduce the lymphangiogenic drive following the initially increased cardiac *Vegfd* expression seen in C57Bl/6 females. In contrast, in Balb/c mice, the ventricular wall stress increased throughout the study, as LV dilation (44% increase in LV ESD at 8 weeks post-TAC *vs* sham) was accompanied by ventricular wall thinning (15–20% reduction in AWT ES and PWT ES, see Supplementary material online, *Table S2*). It thus seems that increased LV wall stress (due to LV dilation), rather than wall thickening (due to cardiac hypertrophy), may be the main trigger for induction of cardiac lymphangiogenesis during pressure-overload. In parallel, a clinical study in HF patients indicated that arterial wall stress, due to elevated pulmonary artery wedge pressure, increased plasma Vegfd levels.^36^

The penetration depth into the myocardium of cardiac lymphatics is species-dependent, with more intramyocardial branches observed in larger animals with thicker walls. For example, previous studies in human and dog hearts^37,38^ indicate that the lymphatic network penetrates the entire myocardium in these species. In contrast, mice, with LV wall thickness of around 1 mm, display lymphatic vessels essentially limited to the cardiac surface also following pressure-overload (see Supplementary material online, *Video S1*). We speculate that this differential species-dependent lymphatic penetration may reflect increased metabolic needs to clear waste products from the cardiac interstitium under settings of elevated wall stress. An additional component that raises wall stress is myocardial oedema, which increases the diastolic stress-strain relationship and decreases cardiac compliance.^39^ However, comparing Balb/c and C57Bl/6 females, we found that the myocardial oedema was similar post-TAC, while our indirect estimates indicated much higher wall stress in the thinning walls of Balb/c mice.

We previously demonstrated that therapeutic lymphangiogenesis limited myocardial oedema post-MI in rats.^6^ It should be noted that following MI there is significant rarefaction and dysfunction of cardiac lymphatics, whereas after pressure-overload cardiac lymphatics were comparably less altered. In particular, we recently demonstrated that poor cardiac lymphangiogenesis post-MI was linked to elevated interferon (IFN)-γ, in part produced by cardiac-infiltrating T cells.^8^ In contrast, previous studies have indicated a predominant *Th2*-type immune response in Balb/c mice, with low cardiac expression of IFN-γ and elevated IL4, leading to DCM in response to chronic arterial hypertension.^15^ Potentially, this immune phenotype of low IFN-γ and elevated IL1β in the heart of Balb/c mice may have been conductive to the robust lymphangiogenic response observed following pressure-overload. However, our preliminary unpublished data, based on treatment with a neutralizing anti-IL1β antibody initiated at 3 weeks post-TAC in Balb/c, revealed no reduction in cardiac lymphangiogenic growth factor expression or lymphangiogenesis, indicating that IL1β is not essential for lymphatic expansion during pressure-overload.

We expected more rapid restoration of myocardial fluid balance post-TAC in Balb/c vs. in C57Bl/6 mice. Thus, our findings that myocardial oedema was similarly severe in all three TAC models, characterized by different degrees of cardiac lymphangiogenesis, was surprising. However, the expansion of essentially capillary lymphatics in Balb/c was clearly not sufficient to restore fluid balance. Moreover, given that we only observed a tendency for an increase in myocardial water content in the anti-VEGFR3-treated TAC group, which suffered cardiac lymphatic rarefaction, we postulate that the main culprit of myocardial oedema in our model may be unrelated to lymphangiogenesis, and instead reflect blood vascular hyperpermeability in the heart resulting from increased coronary blood pressure, and reduced vascular barrier resistance, after aortic banding. Thus, the increased influx of plasma-derived fluids effectively overwhelmed the drainage capacity of cardiac lymphatics, even after potent lymphangiogenesis as in Balb/c. Importantly, this setting is specific for the TAC model, and does not apply in patients with aortic stenosis, who suffer from reduced, rather than increased, perfusion of the coronary vasculature. Thus, it remains to be determined, in a more physiologically-relevant model, whether robust cardiac lymphangiogenesis in response to pressure-overload may be sufficient to improve myocardial fluid balance.

Most recently, a study reported that despite lymphatic expansion following pressure-overload, cardiac lymphatic macromolecular transport capacity was reduced in C57Bl/6 males.^30^ Moreover, the authors demonstrated that cardiac-infiltrating CCR2^+^ Lyve1^neg^ macrophages, by producing matrix metalloproteinase-12, promoted shedding of Lyve1 from cardiac lymphatics. This concords with our findings of reduced *Lyve*1 expression after TAC, and may, together with the noted reduction in lymphatic Vcam-1 expression, explain why, despite massive lymphangiogenesis post-TAC in Balb/c mice, cardiac levels of CD4^+^ T cells and iNOS^+^ macrophages were elevated. Nevertheless, the cardiac levels of other immune cell subsets, including CD206^+^ macrophages, and especially NK cells and B lymphocytes, were much lower at 8 weeks post-TAC in Balb/c as compared with in female C57Bl/6 mice. Taken together, we speculate that while lymphatic fluid transport was not sufficiently improved to prevent myocardial oedema in female Balb/c and male C57Bl/6, their expanded lymphatic networks accelerated clearance of immune cell, as compared with female C57Bl/6 or anti-VEGFR3-treated C57Bl/6 males, both characterized by absence of cardiac lymphangiogenesis at 8 weeks post-TAC. Further investigation of the molecular control of lymphatic trafficking of different immune cell populations in the heart is warranted to better understand the regulation of inflammatory resolution during pressure-overload.

Our data promisingly revealed that even modest cardiac lymphangiogenesis, including periarterial lymphatic expansion, sufficed to limit perivascular fibrosis post-TAC in male C57Bl/6. However, we were surprised by the absence of impact of anti-VEGFR3 treatment on interstitial fibrosis. This differs from our previous findings post-MI in rats, where lymphangiogenesis reduced the development of interstitial fibrosis.^6^ However, one important difference is the degree of cardiac hypoxia and inflammation, both potent drivers of fibrosis,^40^ which both are more severe post-MI as compared with in pressure-overload. Similarly, the apparent absence of impact of expanded lymphatics in Balb/c mice on development of interstitial fibrosis, as compared with in C57B/6 females, may reflect our observation that myocardial oedema was equally severe in both these TAC groups. Of note, during pressure-overload, Balb/c females displayed much higher cardiac expression of pro-fibrotic *Il1b* and *Il6* cytokines, expected to promote cardiac fibroblast activation. Finally, given that ventricular wall stress estimates were higher in Balb/c vs. female C57Bl/6, it seems impertinent to attempt any direct conclusion on how lymphangiogenesis may or may not have altered interstitial cardiac fibrosis between these two strains. Indeed, the metabolic milieu of cardiomyocytes and cardiac fibroblasts was likely very different post-TAC in these two strains due to the difference in wall stress.^41^

Recently, another study reported on the beneficial effects of therapeutic lymphangiogenesis in a mouse TAC model.^42^ The authors surprisingly demonstrate that systemic daily injections of recombinant Vegfc, an inefficient therapeutic modality,^8,10^ potently stimulated cardiac lymphangiogenesis and almost completely prevented myocardial oedema, cardiac hypertrophy and dysfunction during pressure-overload. It should be noted that their study was based on a single-banding TAC method, shown to produce non-permanent constriction in up to 30% of animals.^17^ Although the authors argue that the lower cardiac *Nppa* expression observed after Vegfc therapy was a sign of the cardiac benefit, it is challenging to understand how their systemic therapeutic approach so fully protected against the deleterious effects of pressure-overload as to both prevent and revert LV dilation and hypertrophy. In contrast, our study reveals a subtler functional impact of cardiac lymphangiogenesis during pressure-overload, even in the setting of extensive expansion of the lymphatic network, as in female Balb/c. Indeed, although we speculate that the endogenous lymphangiogenesis may have limited cardiac inflammation and perivascular fibrosis in Balb/c, these mice were not protected against chronic myocardial oedema, interstitial fibrosis, or cardiac decompensation. On the other hand, the moderate lymphangiogenic response, seen in male C57Bl/6 mice post-TAC, was sufficient to limit cardiac inflammation and perivascular fibrosis, and to slow development of cardiac hypertrophy and decompensation.

In conclusion, our findings of more extensive lymphatic remodelling in mice with dilated cardiac phenotype, as well as in HF patients with DCM, indicate that LV dilation, rather than cardiac hypertrophy *per se*, triggers lymphangiogenesis in the heart. Future studies will reveal whether ventricular wall stress is directly linked to lymphangiogenic growth factor expression in the human heart, as suggested by our experimental study, or depends on metabolic alterations in the thinning LV wall. Moreover, while the functional impact of cardiac lymphatic alterations remains to be determined in HF patients, our findings suggest that under settings of pressure-overload poor lymphangiogenesis may accelerate HF development by slowing the resolution of inflammation and aggravating perivascular fibrosis in the heart.

## Supplemental material

Supplementary material is available at *Cardiovascular Research* online.

## Authors’ contributions

C.H., A.D., T.L., M.H., H.C., and O.H. performed and analysed immunohistochemistry and histology. C.H. and A.D. performed and analysed flow cytometry with guidance of G.R. and V.T. Echocardiography was performed and analysed by C.H., M.H., M.D., T.L., and O.H.; microgravimetry was carried out by C.H., O.H., and E.B.; A.L., D.G., D.S., and R.H. carried out, post-processed and analysed light sheet and confocal imaging; J.P.H. and M.V. carried out surgical mouse model; C.H., S.R., and M.H. carried out cardiac gene expression analyses with guidance of S.F.; J.B.M. managed the biobank at Hopital Bichat and contributed clinical data for this study; V.T., P.M., A.O.P., and V.R. participated in manuscript preparation; E.B. and C.H. designed the study, analysed results, and prepared the manuscript draft. All authors approved of the final version of the manuscript.

## Supplementary Material

cvac086_Supplementary_Data

## Data Availability

The data underlying this article are available in the article and in its online supplementary material.
