# Supplementary figures and images for: Regulation and impact of cardiac lymphangiogenesis in pressure-overload-induced heart failure
Source: Cardiovasc Res. 2022 Jun 11;119(2):492–505. doi: 10.1093/cvr/cvac086 (PMC10064842; doi:10.1093/cvr/cvac086)

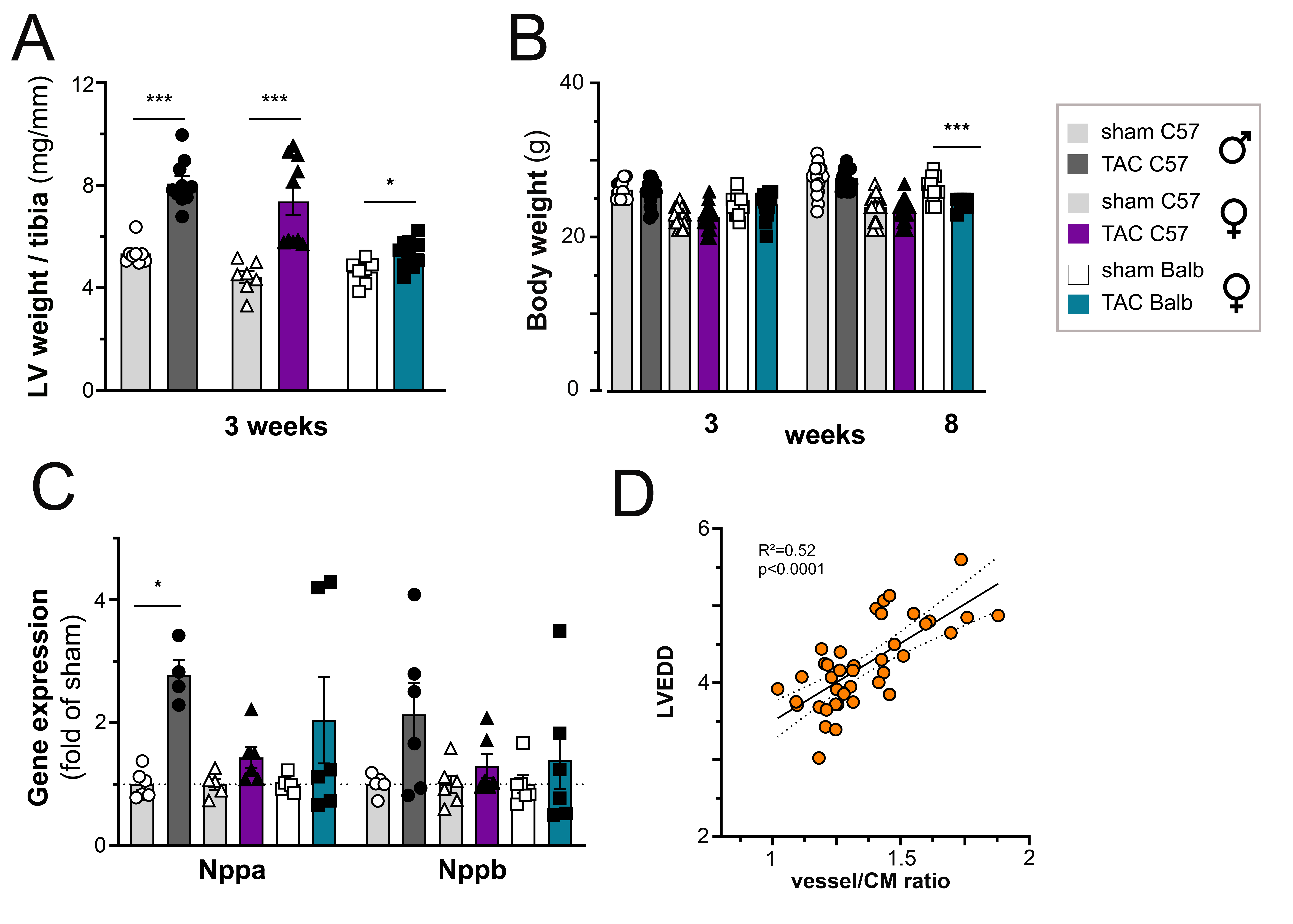

Supplement: cvac086_Supplementary_Data [file cvac086_supplementary_data.zip › Fig S1.tif]

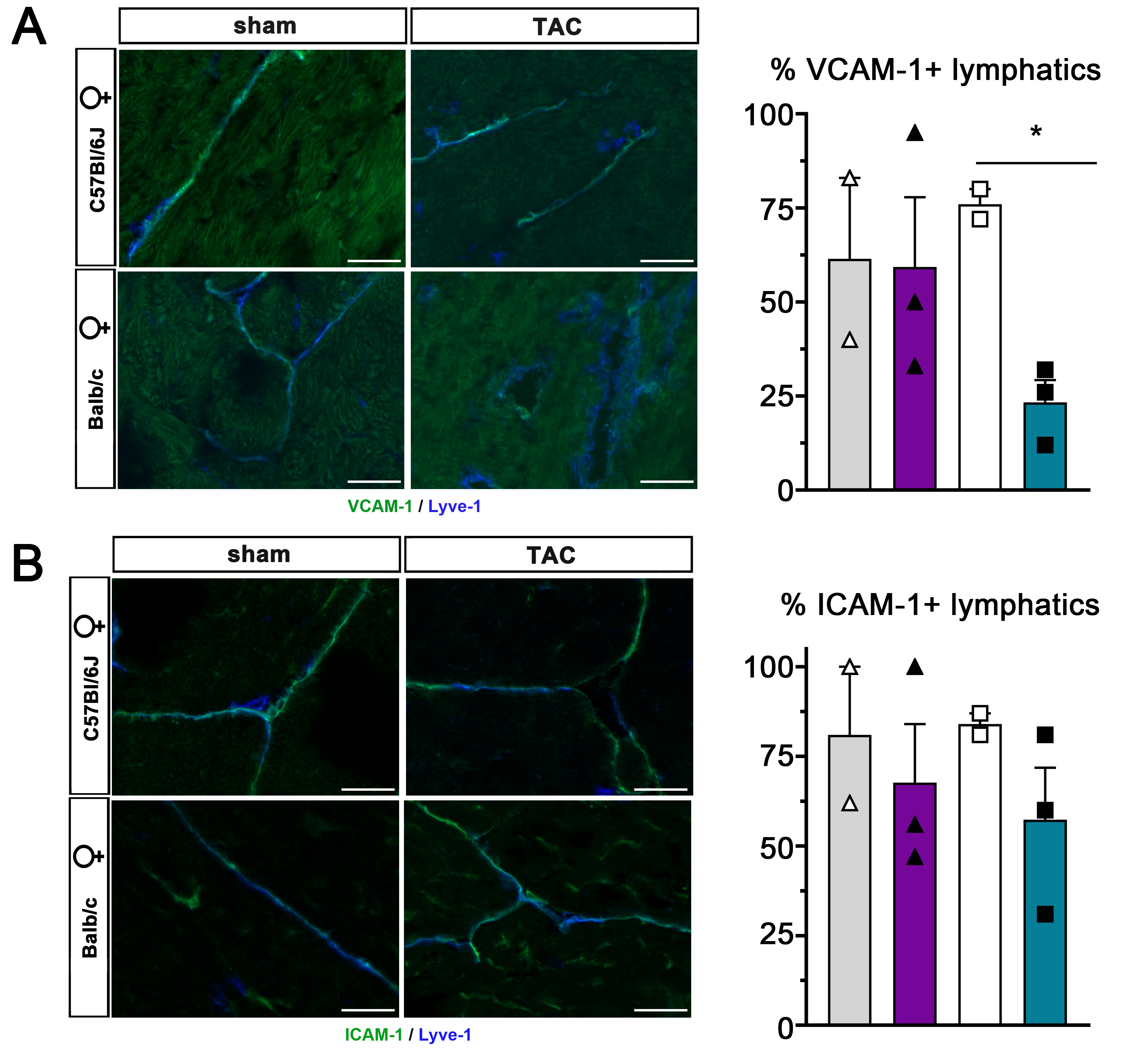

Supplement: cvac086_Supplementary_Data [file cvac086_supplementary_data.zip › Fig S10.tif]

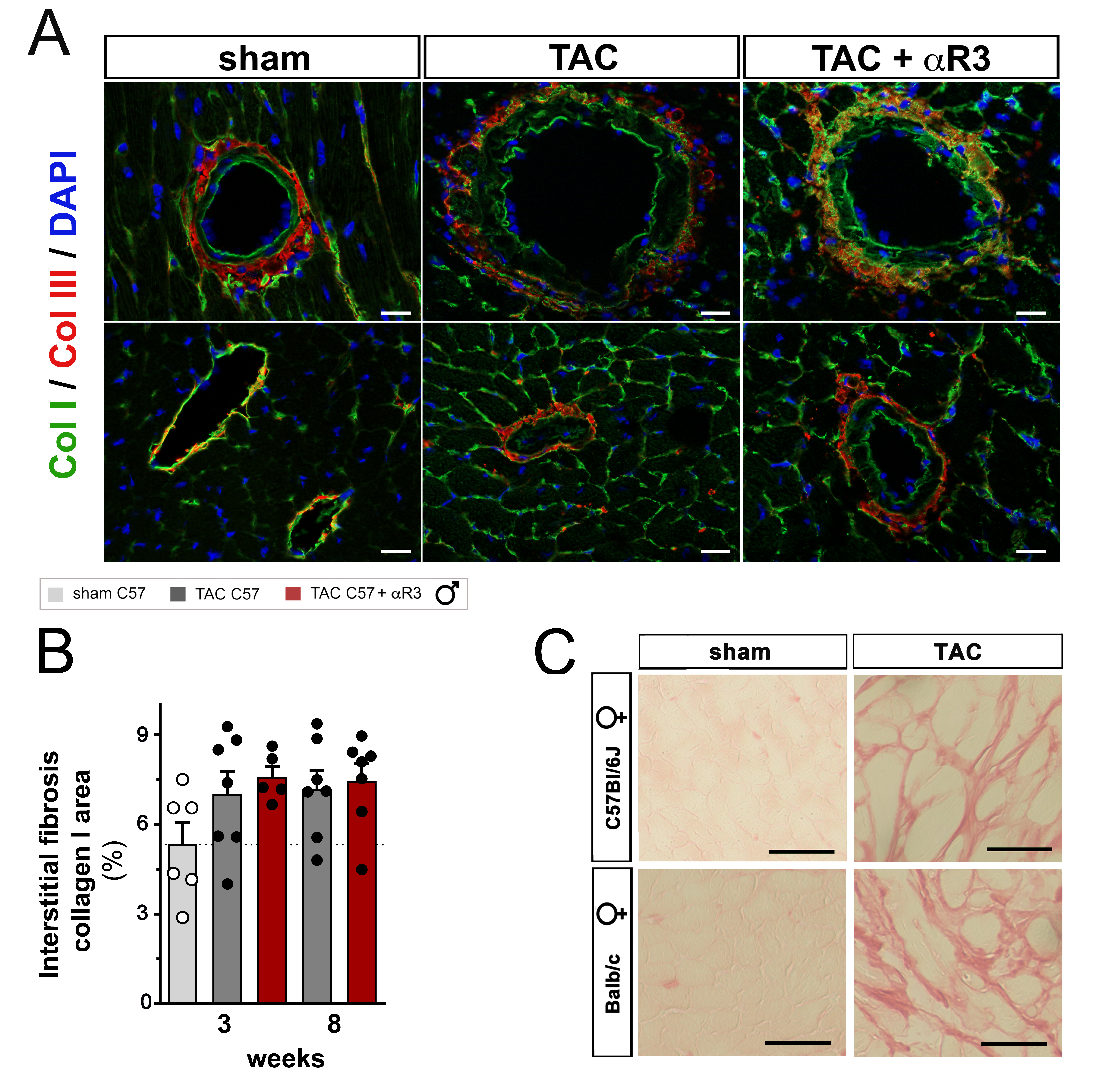

Supplement: cvac086_Supplementary_Data [file cvac086_supplementary_data.zip › Fig S11.tif]

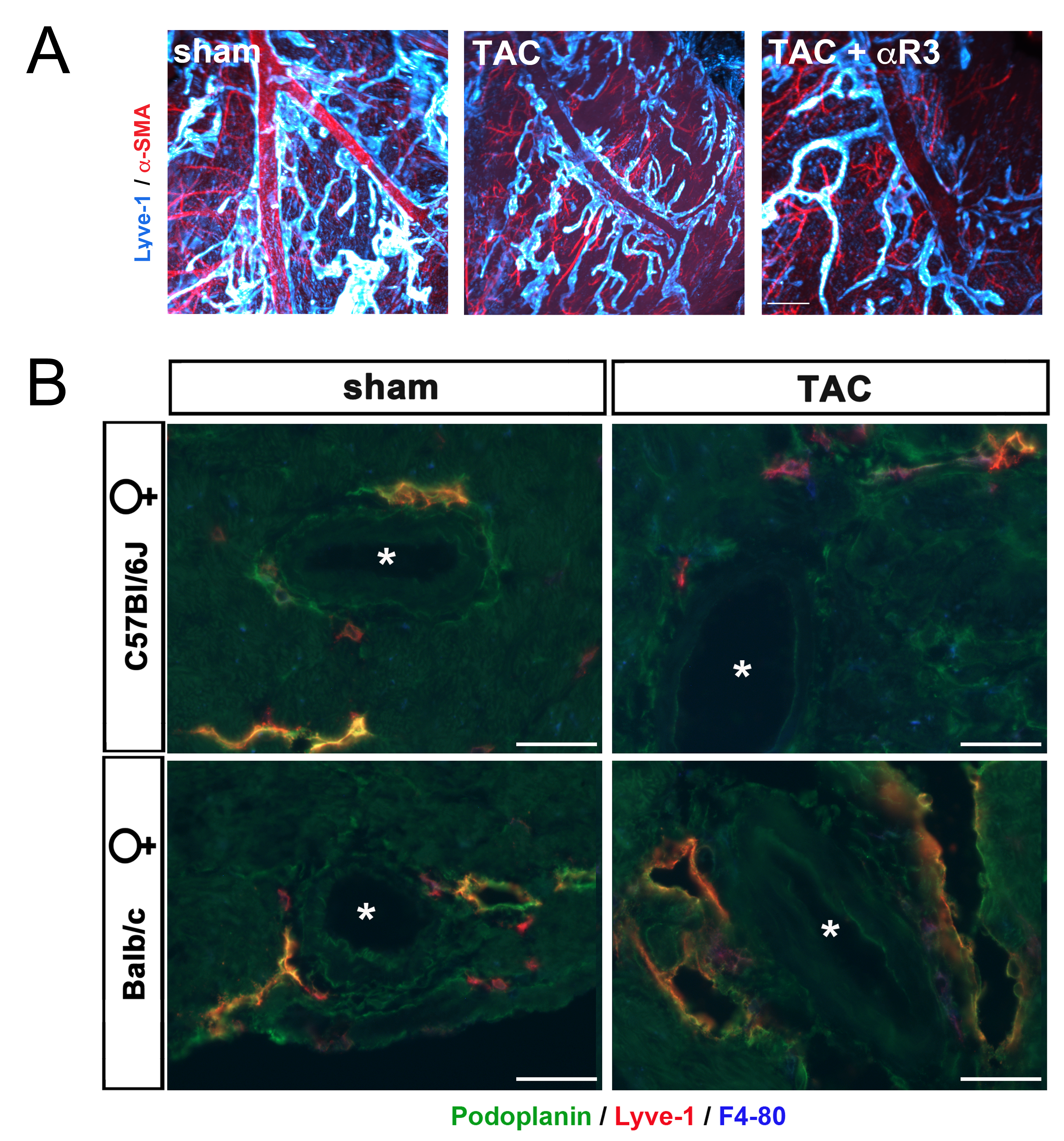

Supplement: cvac086_Supplementary_Data [file cvac086_supplementary_data.zip › Fig S12.tif]

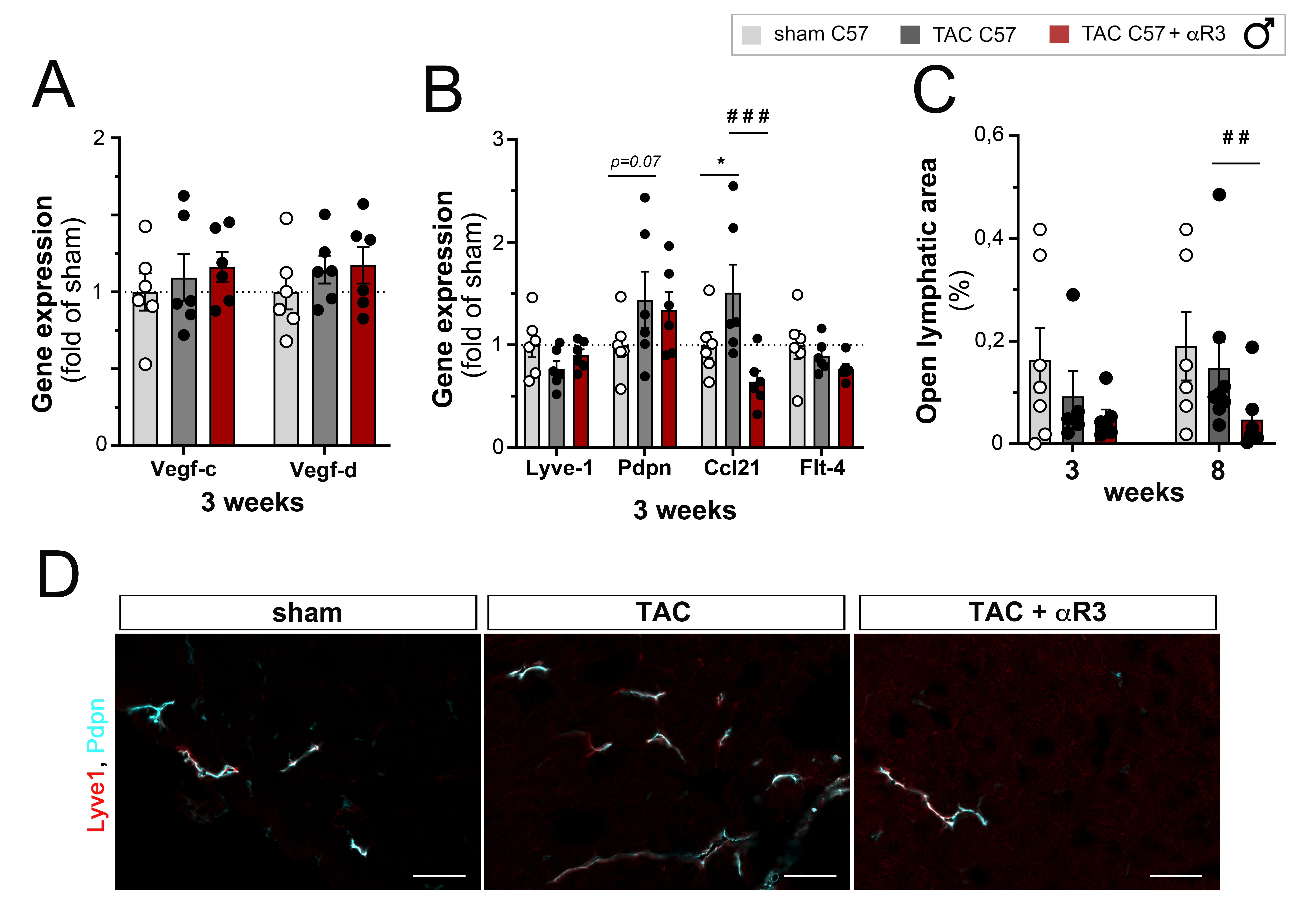

Supplement: cvac086_Supplementary_Data [file cvac086_supplementary_data.zip › Fig S2.tif]

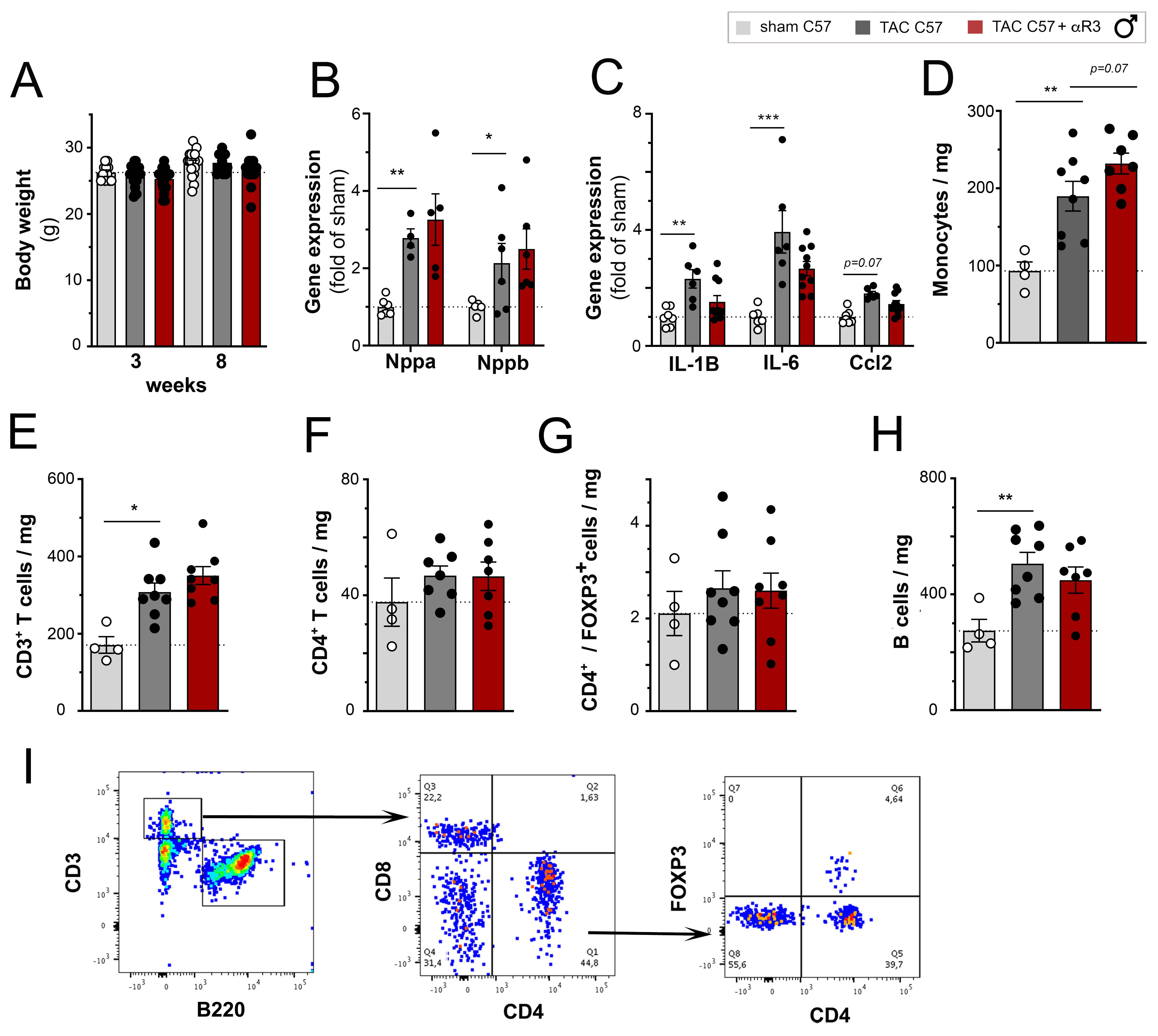

Supplement: cvac086_Supplementary_Data [file cvac086_supplementary_data.zip › Fig S4.tif]

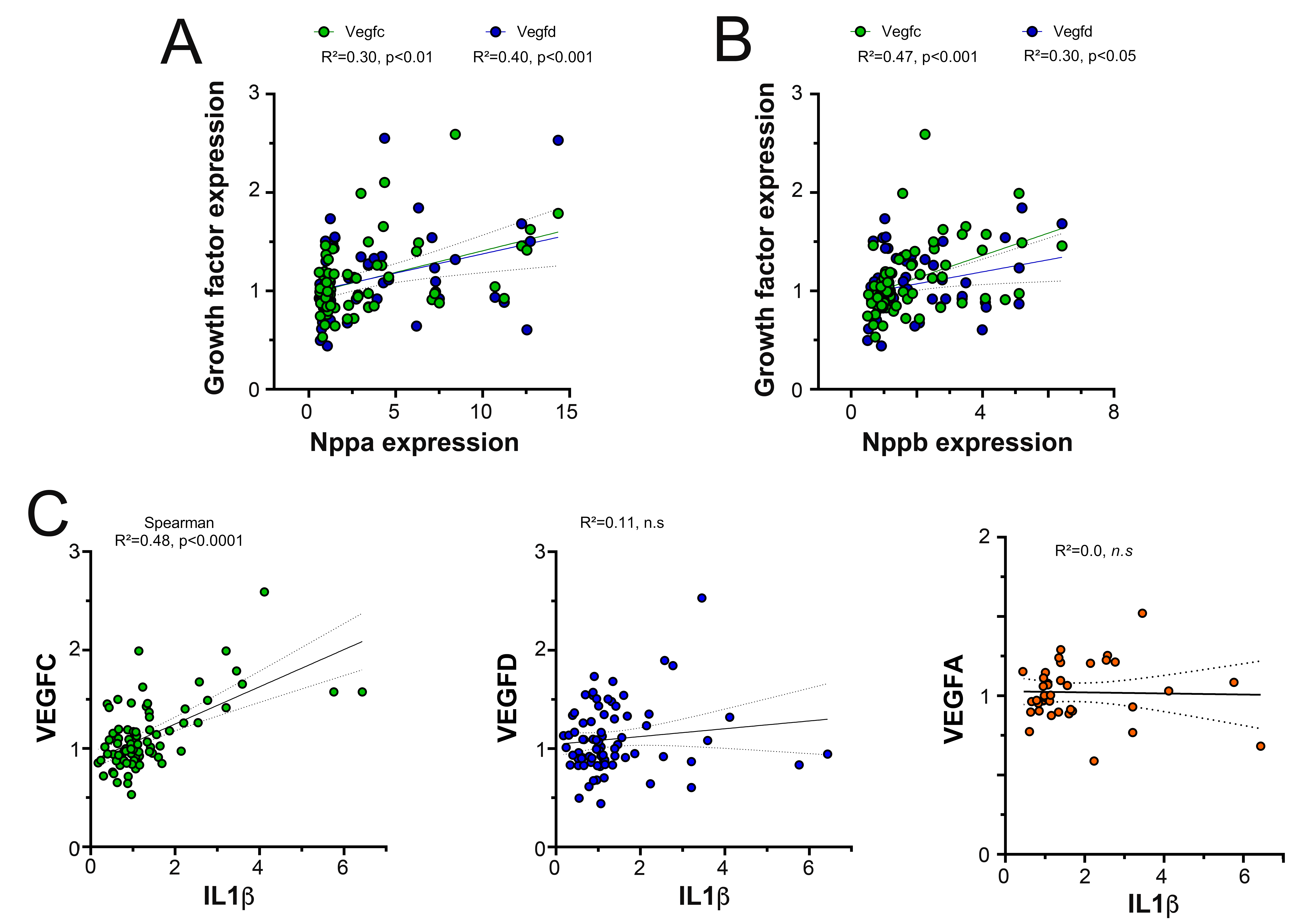

Supplement: cvac086_Supplementary_Data [file cvac086_supplementary_data.zip › Fig S6.tif]

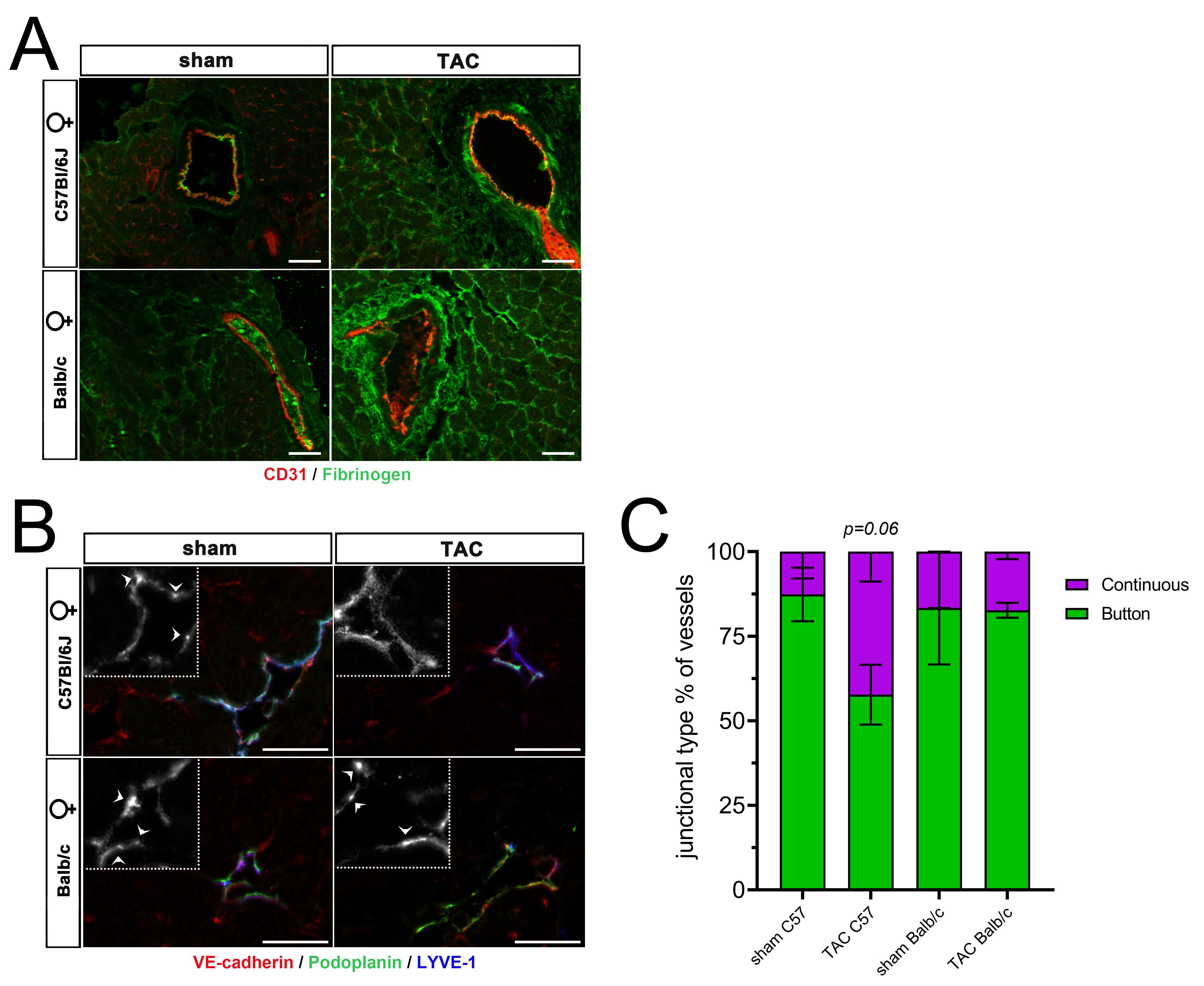

Supplement: cvac086_Supplementary_Data [file cvac086_supplementary_data.zip › Fig S9.tif]
